# Tumor Microenvironment Heterogeneity-Based Score System Predicts Clinical Prognosis and Response to Immune Checkpoint Blockade in Multiple Colorectal Cancer Cohorts

**DOI:** 10.3389/fmolb.2022.884839

**Published:** 2022-06-28

**Authors:** Hufei Wang, Zhi Li, Suwen Ou, Yanni Song, Kangjia Luo, Zilong Guan, Lei Zhao, Rui Huang, Shan Yu

**Affiliations:** ^1^ Department of Colorectal Cancer Surgery, The Second Affiliated Hospital of Harbin Medical University, Harbin, China; ^2^ Department of Obstetrics and Gynecology, The Second Affiliated Hospital of Harbin Medical University, Harbin, China; ^3^ Department of Breast Surgery, Harbin Medical University Cancer Hospital, Harbin, China; ^4^ Department of Gastroenterology, The Second Affiliated Hospital of Harbin Medical University, Harbin, China; ^5^ Department of Pathology, The Second Affiliated Hospital of Harbin Medical University, Harbin, China

**Keywords:** tumor microenvironment, immune checkpoint therapy, colorectal cancer, prognosis, ICB response biomarkers

## Abstract

Despite immune checkpoint blockade (ICB) therapy contributed to significant advances in cancer therapy, only a small percentage of patients with colorectal cancer (CRC) respond to it. Identification of these patients will facilitate ICB application in CRC. In this study, we integrated multiple CRC cohorts (2,078 samples) to construct tumor microenvironment (TME) subtypes using TME indices calculated by CIBERSORT and ESTIMATE algorithms. Furthermore, a surrogate quantitative indicator, a tumor microenvironment immune gene (TMEIG) score system, was established using the key immune genes between TME clusters 1 and 2. The subsequent analysis demonstrated that TME subtypes and the TMEIG score system correlated with clinical outcomes of patients in multiple CRC cohorts and exhibited distinct immune statuses. Furthermore, Tumor Immune Dysfunction and Exclusion (TIDE) analysis indicated that patients with low TMEIG scores were more likely to benefit from ICB therapy. A study on two ICB cohorts (GSE78220 and IMvigor210) also validated that patients with low TMEIG scores exhibited higher ICB response rates and better prognoses after ICB treatment. The biomarker evaluation module on the TIDE website revealed that the TMEIG score was a robust predictive biomarker. Moreover, differential expression analysis, immunohistochemistry, qPCR experiments, and gene set prioritization module on the TIDE website demonstrated that the five genes that constitute the TMEIG score system (*SERPINE1*, *FABP4*, *SCG2*, *CALB2*, and *HOXC6*) were closely associated with tumorigenesis, immune cells, and ICB response indices. Finally, TMEIG scores could accurately predict the prognosis and ICB response of patients with CRC. *SERPINE1*, *FABP4*, *SCG2*, *CALB2*, and *HOXC6* might be potential targets related to ICB treatment. Furthermore, our study provided new insights into precision ICB therapy in CRC.

## Introduction

Colorectal cancer (CRC) is one of the most common malignant tumors globally, with high morbidity and mortality. Tumor immunotherapies involving immune checkpoint blockade (ICB) have contributed to significant advancements in the treatment of many tumors (Topalian et al., 2012), such as melanoma (Luke et al., 2017), bladder cancer (Pettenati and Ingersoll, 2018), and non–small cell lung cancer (Huang et al., 2021). However, most patients with CRC exhibit poor responses to immune checkpoint blockade (ICB) therapy. The biomarkers that predict the efficacy of ICB therapy include the expression of programmed death-ligand 1 (PD-L1) (Nishino et al., 2017), tumor mutation load (Snyder et al., 2014), mismatch repair deficiency (Le et al., 2015), and gut microbiota (Daillère et al., 2016; Routy et al., 2018). However, there is still a lack of effective tools to predict the ICB response in CRC, which impedes the application of ICB therapy in CRC. Therefore, there is an urgent need to establish effective and reliable tools for predicting response to ICB therapy and achieving precision therapy in patients with CRC.

The tumor microenvironment (TME) mainly contains tumor, immune and stromal cells, and small molecules (Vitale et al., 2019). TME of CRC exhibits remarkable heterogeneity (Zhang et al., 2020), which can cause variation in tumor biology, thus affecting the efficacy of multiple therapies (Casey et al., 2015; Wu and Dai, 2017), including chemotherapy (Hanoteau et al., 2019), radiotherapy (Yin et al., 2019; Sheng et al., 2020), and immune checkpoint therapy (Lei et al., 2020; Sheng et al., 2020). In addition, the TME can predict the prognosis of patients with CRC (Pagès et al., 2005). For example, a high M1:M2 density ratio in tumor stroma was associated with better cancer-specific survival (Väyrynen et al., 2021). Immune cells in the TME play critical roles in the efficacy of immunotherapy (Arce Vargas et al., 2018; Väyrynen et al., 2021). Patients with higher CD8 cells in the TME exhibit more favorable responses to ICB (Pagès et al., 2005). T cell-dendritic cell crosstalk facilitates successful anti–PD-1 immune therapy (Zhao et al., 2019). Therefore, studying TME heterogeneity will help reveal the biological characteristics of CRC, assist the implementation of precision therapy, and guide the application of ICB. However, the TME is an extremely complex system. It is critical to establish a simple surrogate gene model of the TME to predict the prognosis of patients and the efficacy of ICB therapy.

In the present study, we integrated transcriptome data of 1,175 patients with colorectal cancer from the GPL570 platform and then employed CIBERSORT, ESTIMATE, and ssGSEA algorithms to assess the characteristics of the TME. Based on TME heterogeneity, two TME subtypes (clusters 1 and 2) with different survival statuses were identified using a consensus clustering algorithm. Weighted gene co-expression network analysis (WGCNA), linear models for microarray data (LIMMA), and other bioinformatics analyses were used to identify the hub TME immune genes between subtypes. Then, patients were also divided into two tumor microenvironment immune gene (TMEIG) subtypes (clusters A and B) with different survival statuses according to the hub TME immune genes. Moreover, a robust prognostic scoring system (TMEIG score) was developed using these TME immune genes, which could effectively predict overall survival (OS), progression-free survival (PFS), and disease-specific survival (DSS) of patients with CRC. The prognostic TMEIG score system was also verified in multiple cohorts, such as TCGA-COAD. Notably, we validated that the TMEIG score could predict ICB response in multiple immunotherapy cohorts and is expected to guide the application of ICB in CRC.

## Materials and Methods

### Data Source and Process

Ten data sets were downloaded from the public database, including clinical data and transcriptome data of 2,078 patients with CRC. GSE39582, GSE14333, GSE17536, GSE17537, and GSE72968 were all microarray data of the GPL570 platform and integrated as a training set (the combined GEO cohort). The CEL format data of the microarray was downloaded using the “GEOquery” package (Davis and Meltzer, 2007). ReadAffy function in the “affy” package was used to read data in CEL format (Gautier et al., 2004), background correction and standardization were carried out with RMA, and then the “SVA” package was used to remove batch effect among the data sets (Chakraborty et al., 2012). Probes corresponding to multiple genes were deleted, and the average expression level was taken when multiple probes corresponded to one gene. Clinical data and FPKM value (fragments per kilobase million) of TCGA-COAD were obtained from the UCSC website as an external validation set (Navarro Gonzalez et al., 2021). Then, FPKM was converted to TPM (transcripts per kilobase million) for subsequent analysis. GSE39582, GSE17536, and GSE17537 were used as the internal validation sets. The paired samples of GSE44076, GSE32323, GSE89076, and GSE113513 datasets have been reserved for verifying the gene expression level in the TMEIG score system. Detailed information on the data sets is shown in Supplementary Table S1.

### Characteristics of the Tumor Microenvironment

CIBERSORT (Newman et al., 2015) and ESTIMATE (Becht et al., 2016) algorithms can infer the composition of 22 types of immune cells, immune score, stroma score, and tumor purity in the TME based on transcriptome data. In this study, transcriptome data from the combined GEO dataset (1,175 samples) and TCGA COAD dataset (471 tumors) were used for CIBERSORT and ESTIMATE analyses. After filtering out low expression immune cells, 15 types of immune cells were retained. ssGSEA is another algorithm for estimating immune cell composition in solid tumor TME and can also be used to calculate adaptive and innate immune components of samples. In the present study, the adaptive and innate immune scores of each sample were obtained with the “GSVA” package. ssGSEA parameters were set as follows: method = “ssgsea,” KCDF = “Gaussian.”

### Unsupervised Clustering

“ConsensusClusterPlus” is a re-sampling unsupervised clustering method to verify the rationality of clustering (Wilkerson and Hayes, 2010). In the combined GEO cohort, TME subtypes and TMEIG subtypes were obtained using “ConsensusClusterPlus” package. The parameters were set as follows: MaxK = 9, REPS = 1,000, pItem = 0.8, pFeature = 1, clusterAlg = “PAM,” distance = “Euclidean,” and seed = 1.

### Weighted Gene Co-Expression Network Analysis

WGCNA analysis was used to identify gene modules most associated with traits (Langfelder and Horvath, 2008). Stromal score, immune score, estimate score, tumor purity, adaptive immune, innate immune, TME cluster 1, and TME cluster 2 were inputted as traits. The key parameters of WGCNA were set as follows: soft threshold power β = 4, scale-free R2 = 0.89. The relationship between modules and traits was analyzed using the Pearson correlation method. Gene significance (GS) and module membership (MM) are two important indicators in WGCNA analysis. GS is the correlation between the gene and trait. MM is defined as the correlation of the module eigengene and the gene expression profile. Genes with GS > 0.2 and MM > 0.8 are usually considered hub genes.

### Functional Enrichment Analyses

Gene Ontology (GO) and Kyoto Encyclopedia of Genes and Genomes (KEGG) (Kanehisa and Goto, 2000) analyses were employed to explore the biological functions of the modules in WGCNA using the R package “clusterprofiler” (Yu et al., 2012). An adjusted *p*-value of less than 0.05 was regarded as statistically significant. In addition, Gene Set Enrichment Analysis (GSEA) was conducted (Subramanian et al., 2005). The gene sets “c2.cp.kegg.v6.2.symbols.gmt,” “c5.all.v7.0.symbols.gmt,” and “h.all.v7.2.symbols.gmt” on MSigDB website were chosen as the reference gene sets (Liberzon et al., 2015). The log fold change (FC) of differentially expressed genes between two groups was used as the input list for GSEA analysis. When analyzing the biological functions related to one gene, the Pearson correlation coefficient was used as the input list.

### Construction and Validation of TMEIG Score

First, univariate Cox proportional hazards regression was employed to identify the prognostic genes using the “survival” R package. Genes with a *p*-value less than 0.05 were regarded as the candidates, input to least absolute shrinkage and selection operator (LASSO) regression (Friedman et al., 2010). After ten cross-validations, five prognostic genes and the corresponding coefficient remained when lambda = 0.0713387182. Then, TMEIG score was established as follows:

$$\mathrm{TMEIG score}=\sum_{i}\mathrm{Coefficient of}\left(i\right)\times \mathrm{Expression of gene}\left(i\right)$$

The regression coefficient of the gene was designated (i) in the LASSO regression model. The combined GEO cohort was used as a training set, whereas GSE39582, GSE17536, and GSE17537 were used as the internal validation sets. In addition, the TCGA COAD cohort served as the external validation set.

### Survival Analysis

Only GSE39582, GSE17536, GSE17537, and GSE72968 had overall survival data in the combined GEO cohort (Supplementary Table S2). The survival time was converted to months, and samples with a survival time of less than 1 month were removed during survival analysis. Finally, 864 samples in the combined GEO cohort and 435 samples in the TCGA COAD cohort were used for survival analysis (Supplementary Table S2). According to the best cutoff value determined using the “survminer” package, the patients were divided into high and low expression groups. Log-rank test was employed to evaluate statistical significance. Kaplan–Meier (KM) plots were visualized using the “survminer” R package. The risk factors diagrams were visualized using the “ggrisk” R package.

### Analysis of Mutation Data

The mutation data of TCGA COAD were downloaded from the TCGA website and analyzed using the “maftools” package (Mayakonda et al., 2018). The tumor mutation burden (TMB) was calculated using the following formula: (total mutation/total covered bases) × 106. The driver genes in somatic alterations were also identified using the “maftool” package.

### ICB Response Prediction

Tumor Immune Dysfunction and Exclusion (TIDE) algorithm was employed to predict ICB response based on the gene expression related to T cell dysfunction (Dysfunction) and T cell exclusion (Exclusion). The lower the TIDE score is reportedly associated with a better immunotherapy response (Jiang et al., 2018). Furthermore, the scores of cancer-associated fibroblasts (CAF), Dysfunction, Exclusion, M2 macrophages (M2), myeloid-derived suppressor cells (MDSC), and TIDE were obtained from the TIDE website. The IMvigor210 cohort is a large cohort of patients with metastatic urothelial cancer under anti–PD-L1 therapy (atezolizumab), which can be downloaded from the Creative Commons 3.0 license. GSE78220 is an anti–PD-1 therapy cohort containing mRNA expression data from pre-treatment melanomas. The two cohorts were used to validate the predictive power of the TMEIG score for ICB response.

### Cell Culture

The human CRC cell lines SW620, RKO, HCT116, HT29, and NCM460 (ATCC) were cultured in RPMI-1640 medium (Gibco, United States ) supplemented with 10% fetal bovine serum (FBS, Biological Industries, United States ) at 37°C in a humidified 5% CO_2_ atmosphere.

### RNA Extraction and Quantitative Real-Time PCR

Total RNAs of cell lines were extracted by TRIzol reagent (Invitrogen, United States ) and then was reversely transcribed as cDNA *via* PrimeScript™ RT Master Mix (Takara, Japan). Quantitive real-time PCR was performed using PowerUp™ SYBR™ Green Master Mix (Applied Biosystems, United States) in the StepOne™ Real-Time PCR System (Applied Biosystems). Each reaction was tested in triplicate. ACTB was used as the internal reference, and the 2(^−ΔΔCT^) method was used for calculating the relative mRNA expression. The following primer sets were used:

Human *FABP4*: Forward: 5ʹ-GGG​CCA​GGA​ATT​TGA​CGA​AG-3ʹ, Reverse: 5ʹ-TCG​TGG​AAG​TGA​CGC​CTT​TC-3ʹ; Human *SCG2*: Forward: 5ʹ-GTG​AAG​CGA​GTT​CCT​GGT​CA-3ʹ, Reverse: 5ʹ-ATG​CTC​TTT​GAT​GGC​CTG​CT-3ʹ; Human *CALB2*: Forward: 5ʹ-GAA​GGC​AAG​GAA​AGG​CTC​TGG-3ʹ, Reverse: 5ʹ-GCC​ATC​TCG​ATT​TTC​CCA​TCT​G-3ʹ; Human *SERPINE1*: Forward: 5ʹ-CCT​GGT​TCT​GCC​CAA​GTT​CT-3ʹ, Reverse: 5ʹ-CCA​TGC​GGG​CTG​AGA​CTA​TG-3ʹ; Human *HOXC6*: Forward: 5ʹ-CAC​TAA​CCC​TTC​CTT​ATC​CTG​CC-3ʹ, Reverse: 5ʹ-TCA​TAG​GCG​GTG​GAA​TTG​AGG-3ʹ; Human *ACTB*: Forward: 5ʹ-GAT​TCC​TAT​GTG​GGC​GAC​GA-3ʹ, Reverse: 5ʹ-AGG​TCT​CAA​ACA​TGA​TCT​GGG​T-3ʹ.

### Immunohistochemistry

For the IHC experiment, we collected 16 pairs of CRC tissue (cancer and adjacent normal tissue) from patients who received surgery at the Department of Colorectal Cancer Surgery, the Second Affiliated Hospital of Harbin Medical University (Harbin, China) between January 2014 and December 2020. Ethics approval was also granted by the Ethics Committee of Harbin Medical University (No. KY 2022-063). The primary antibodies used in IHC were as follows: anti-FABP4 (Proteintech, #12802-1-AP, 1:200 dilution), anti-SCG2 (Proteintech, #20357-1-AP, 1:200 dilution), anti-CALB2 (Proteintech, #12278-1-AP, 1:200 dilution), anti-HOXC6 (Affinity, #DF3078, 1:150 dilution), and anti-PAI1 (SERPINE1) (Affinity, #AF5176, 1:200 dilution). Paraffin sections were incubated with primary antibodies at 4°C overnight, followed by treatment with HRP-conjugated secondary antibodies at 37°C temperature for 60 min following PBS rinse. Then, tissues were counter-stained with hematoxylin and further treated with DAB for 2 min. The IHC results were independently analyzed by two experienced pathologists. A staining scoring system was evaluated by both staining intensity (negative = 0, weak = 1, and strong = 2) and staining area (<5% = 0, 5%–25% = 1, 25%–50% = 2, 50%–75% = 3, and >75% = 4). The staining intensity score was computed, and the score of the staining area was the final staining score. A total score of ≤3 was considered a weak expression. A total score of >3 was considered a strong expression. The details of IHC performance and scoring system are described in Supplementary Table S3.

### Statistical Analysis

Heat maps were visualized with the “ComplexHeatmap” package (Gu et al., 2016). The “ggplot2” package was used to visualize boxplots, scatter plots, and Sankey plots. The log-rank test and Pearson method were used for KM survival and correlation analyses, respectively. The difference between the two groups was tested by the Wilcox test. It should be noted that * represented a *p*-value less than 0.05, ** represented a *p*-value less than 0.01, *** represented a *p*-value less than 0.001, and **** represented a *p*-value less than 0.0001. All analyses were performed in R 4.0.3.

## Results

### Depicting the Heterogeneity of the Tumor Microenvironment in a Large CRC Cohort

**FIGURE 1:**
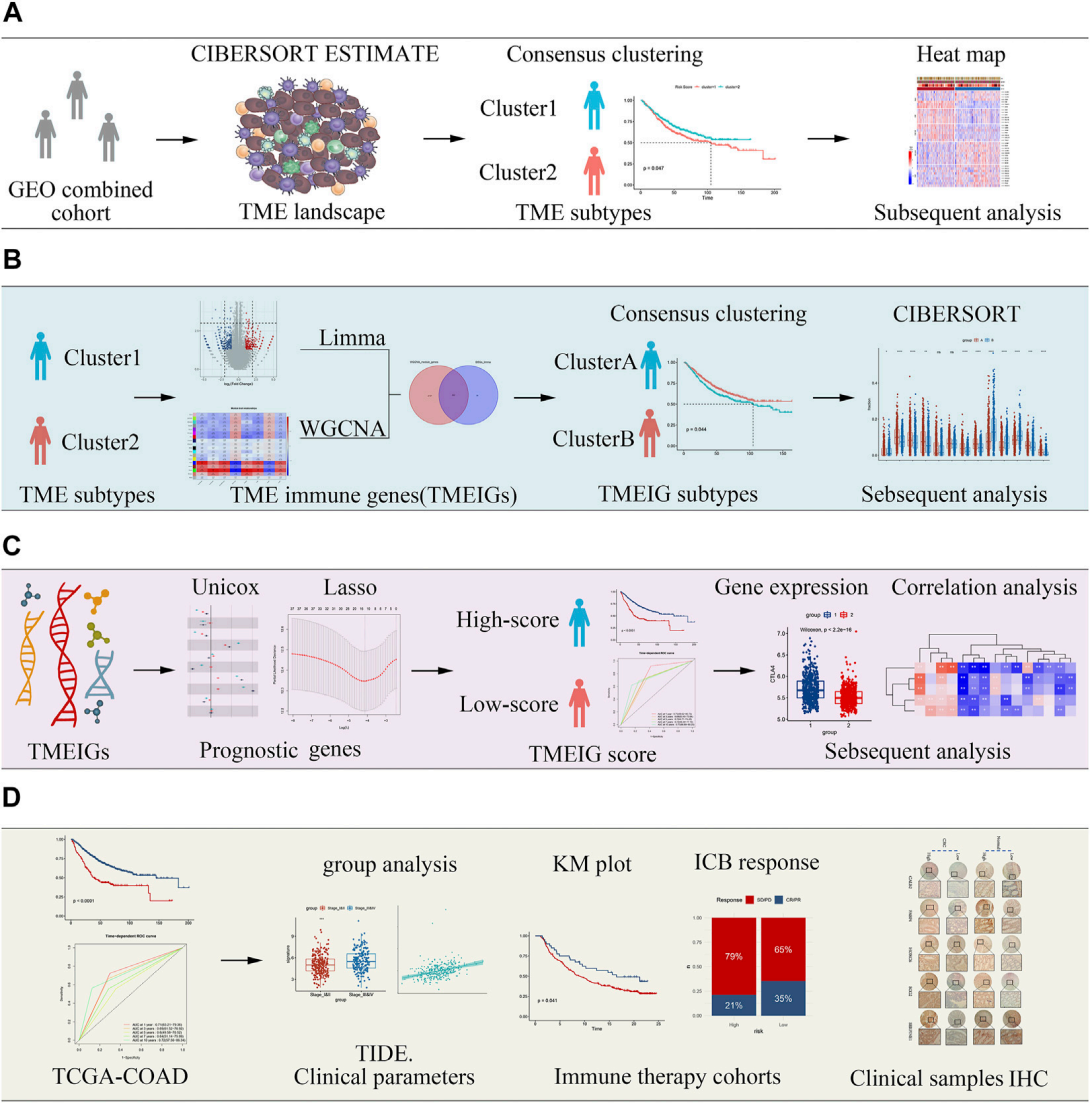
Flow diagram of the study describing the process by which tumor microenvironment (TME) subtypes and the key tumor microenvironment immune gene (TMEIG) scoring system were identified. **(A)** Identification of TME subtypes. **(B)** Construction of TMEIG subtypes. **(C)** Establishment of TMEIG score. **(D)** Validation of TMEIG score in immune therapy cohorts.

**FIGURE 2:**
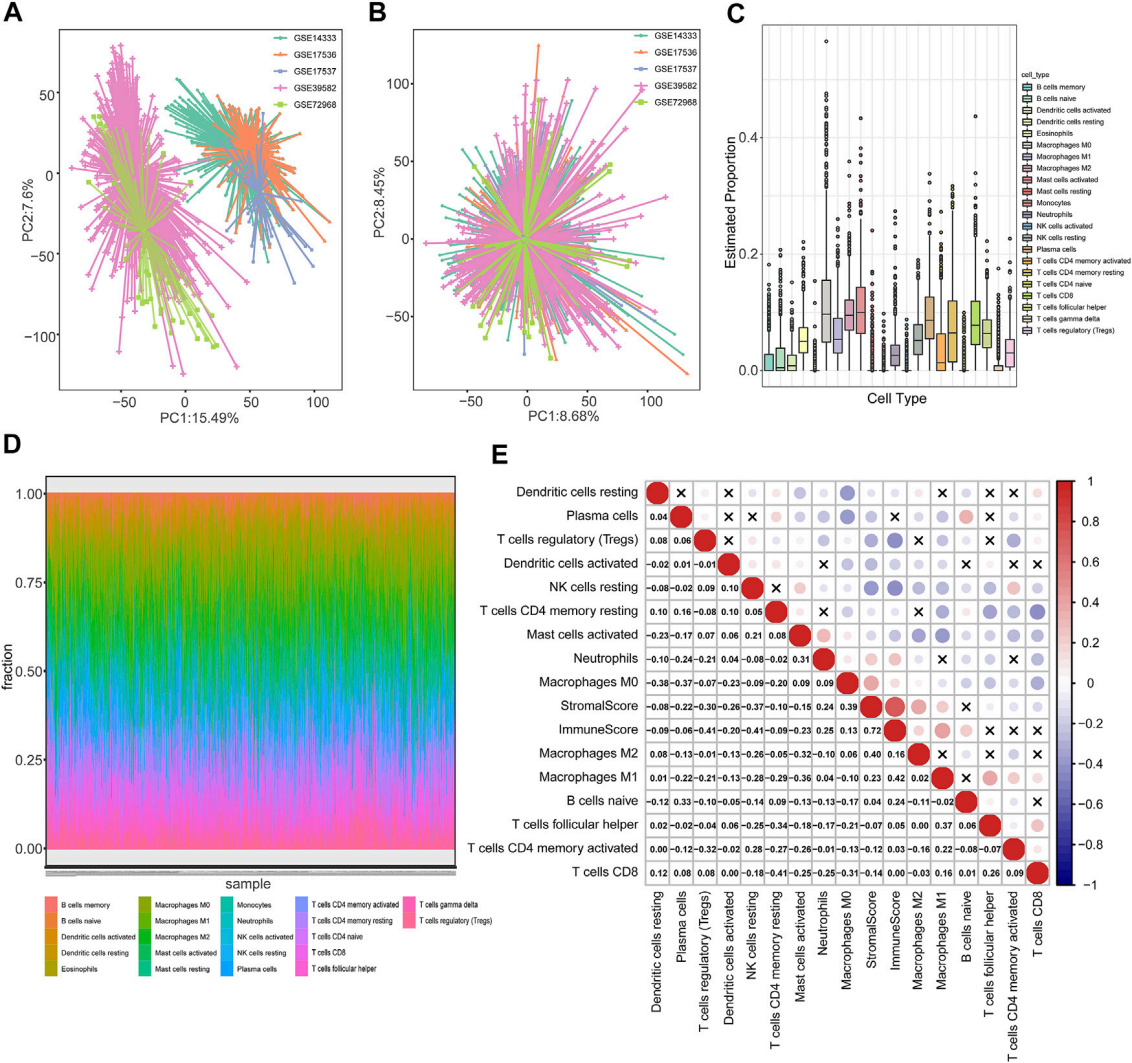
Heterogeneity of the TME in patients with colorectal cancer. **(A,B)** PCA diagrams of five cohorts (GSE39582, GSE14333, GSE17536, GSE17537, and GSE72968) before and after batch effect removal. **(C)** Boxplot cells, immune score, and stromal score (calculated using the ESTIMATE algorithm) in TME of patients with CRC were analyzed using the “corrplot” R package. Red and blue colors represent positive and negative, depicting the proportion of 22 types of immune cells in TME estimated using the CIBERSORT algorithm. **(D)** The distribution of 22 types of immune cells in each patient. **(E)** Pearson correlation between immune correlations, respectively. The correlation *p*-values were less than 0.05 in all cases except those marked with “x” symbols.

The flow diagram describes the construction of TME subtypes and the TMEIG score in CRC (Figure 1). We integrated microarray data of 1,175 patients with CRC from the GPL570 platform and then used the combat function of the “SVA” package to remove batch effects. The principal component analysis (PCA) diagrams of five cohorts before and after batch effect removal are shown in Figures 2A,B. The results indicated that the batch effect was negated, and the combined cohort could be used for subsequent analysis. To fully dissect the heterogeneity of the TME in patients with CRC, the CIBERSORT algorithm was used to assess the proportion of immune cells in the TME. Macrophages and mast cells were the most abundant immune populations in the combined GEO cohort, followed by memory resting CD4 and CD8 T cells. Figures 2C,D shows the proportion of immune cells in each patient, which partly reflects the heterogeneity of immune cells in the TME. A total of 15 types of immune cells were retained after eliminating low expression cells (such as memory B cells, CD4 naive T cells, gamma delta T cells, activated NK cells, monocytes, resting mast cells, and eosinophils). The detailed results of the CIBERSORT analysis are shown in Supplementary Table S4. Then, the ESTIMATE algorithm was used to calculate patients’ immune scores and stromal scores. Collectively, CIBERSORT and ESTIMATE algorithms were used to comprehensively describe the correlations among the immune cells, immune score, and stromal score in the tumor microenvironment of patients with CRC (Figure 2E). Resting NK cells were inversely correlated with M0/M1/M2 macrophages (correlation values = −0.09, correlation values = −0.28, correlation values = −0.26; *p*-value < 0.05; Supplementary Table S4). Furthermore, CD8 T cells were negatively related to M0 macrophages (correlation values = −0.31, *p*-value < 0.05) and positively related to M1 macrophages (correlation values = 0.16, *p*-value < 0.05).

### Tumor Microenvironment Cluster 2 has Better Survival and Exhibits a Different Immune State

**FIGURE 3:**
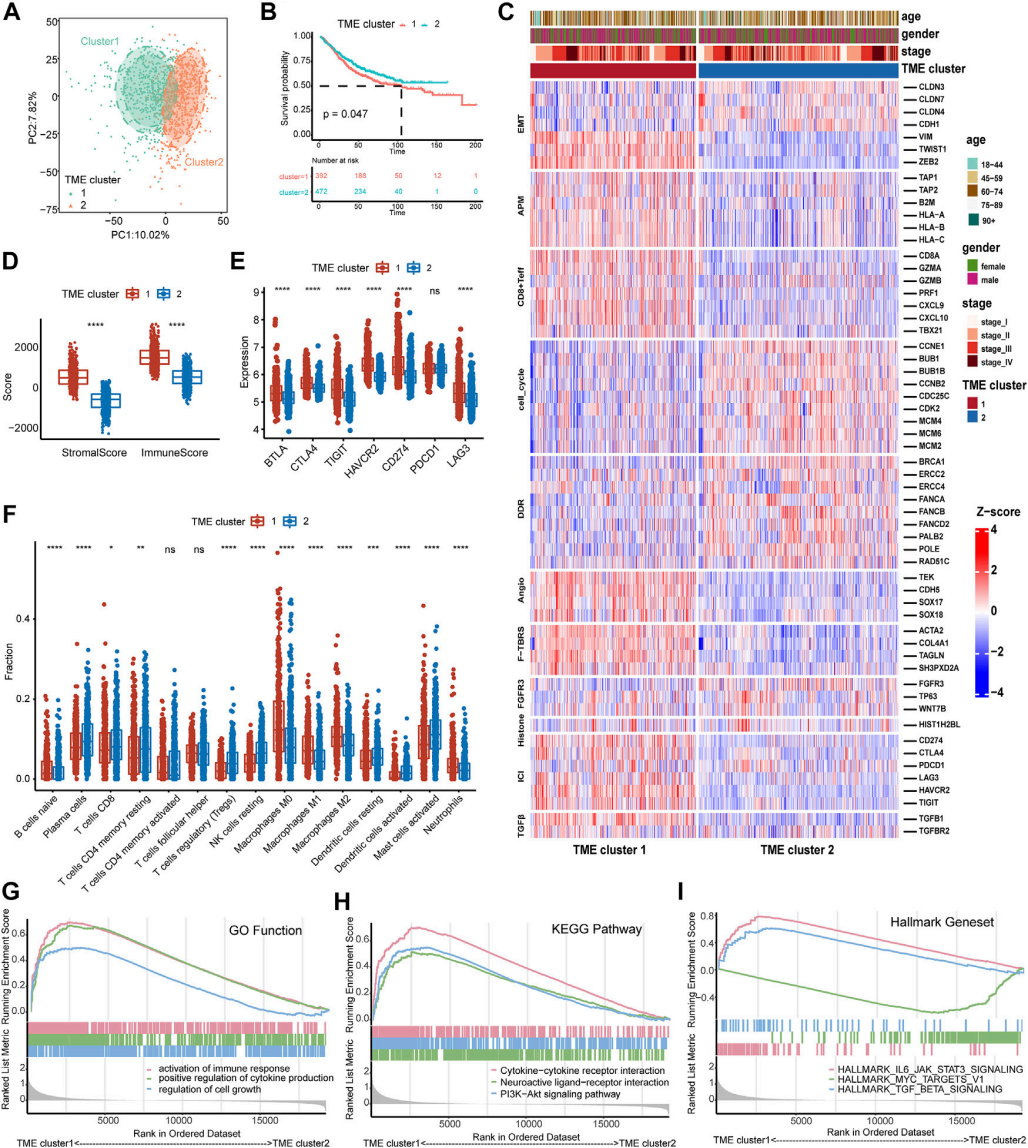
Identification of the TME subtypes and analysis of biological functions. **(A)** PCA analysis demonstrates that the TME subtypes display distinct gene expression signatures. **(B)** The survival analysis of TME subtypes in the combined GEO cohort. **(C)** Relationship between TME subtypes and 11 critical biological pathways. Rows of the heat map represent gene expression grouped by pathway. Red and blue colors represent high and low expression, respectively. EMT (epithelial to mesenchymal transition), Angio (angiogenesis), ICI (immune checkpoint genes), DDR (DNA damage-repair), and APM (antigen-processing machinery). **(D)** The boxplot of the immune score and stromal score between TME subtypes calculated by ESTIMATE analysis. **(E)** The boxplot of seven immune checkpoint genes between TME subtypes. **(F)** The distribution of 15 types of immune cells between TME subtypes estimated by CIBERSORT analysis. **(G–I)** GSEA analysis of GO function, KEGG pathway, and Hallmark gene set of both TME subtypes. The difference between the two groups was assessed using the Wilcox test. The log-rank test was used for KM survival analysis.

Based on these quantitative indicators describing the TME, we conducted unsupervised clustering in these 1,175 patients using the “ConsesusClusterPlus” package. As shown in Supplementary Figure S1, the clustering result was the most stable when *K* = 2. The PCA plot also demonstrated significant differences between the two clusters (Figure 3A). Then, survival analysis was employed to compare the prognosis between the two TME clusters (Supplementary Table S5). The OS in TME cluster 2 was significantly better than that in TME cluster 1 (Figure 3B log-rank test, *p* = 0.047). Furthermore, we explored 11 critical biological gene signatures between the two TME subtypes using a heat map (Mariathasan et al., 2018). The results indicated that cell cycle genes and DNA damage repair (DDR) genes were markedly decreased, and angiogenesis (Angio) markers, TGFβ receptor and ligand (TGFβ), antigen-processing machinery (APM), and F-TBRS genes were significantly increased in TME cluster 1 as compared to TME cluster 2 (Figure 3C). In addition, CD8 Teff cells and immune checkpoint signatures (ICI) were highly expressed in TME cluster 1 (Figures 3C–E). The low expression of cell cycle-associated genes may indicate that tumor cells in TME cluster 1 were in a dormant phase and were not easily cleared by the immune system. A comparison of the immune score and stromal score revealed that the immune score and stromal score of TME cluster 1 were higher than those of TME cluster 2 (Figure 3D). CIBERSORT analysis demonstrated that immunosuppressive cells (M0, M1, and M2) were significantly reduced, and immunoreactive cells (CD8 T cell, CD4 memory resting T cells, resting dendritic cells, and activated dendritic cells) were significantly increased in the TME cluster 2 (Figure 3F). Furthermore, the GSEA results indicated that immune-related functions (activation of immune response, positive regulation of cytokine production, cytokine–cytokine receptor interaction, and IL6-JAK-STAT3 signaling) significantly varied between TME clusters 1 and 2 (Figures 3G–I; Supplementary Table S6).

### Identification of Key Tumor Microenvironment Immune Genes Between Tumor Microenvironment Subtype

**FIGURE 4:**
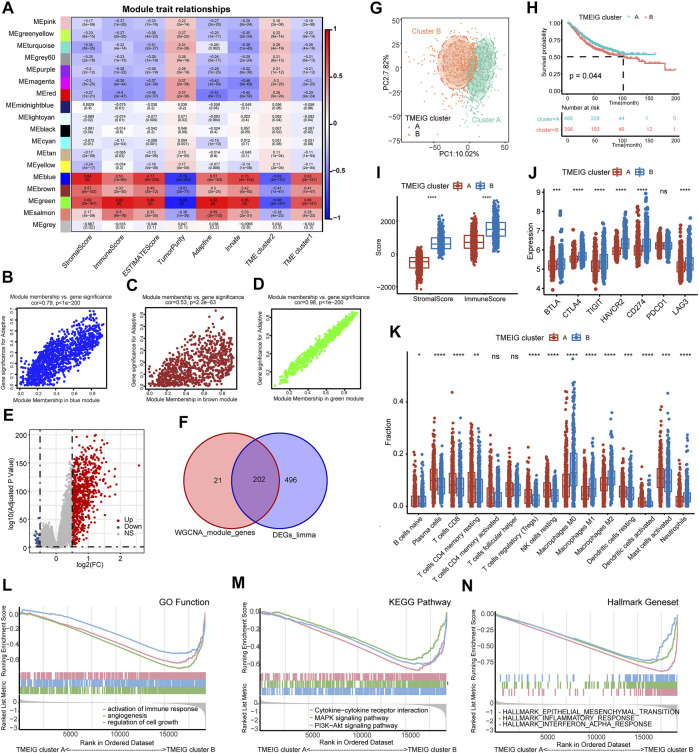
Identification of TMEIG and TMEIG subtypes. **(A)** Heatmap of module trait relationships in the combined GEO cohort. Each row contains the corresponding correlation values and *p*-value. Red and blue colors represent the positive and negative correlations, respectively. **(B–D)** Scatter plots of the correlation between module eigengenes and adaptive immune in blue, brown, and green modules. **(E)** The volcano plot of the differentially expressed genes between TME clusters 1 and 2. **(F)** The intersection genes of WGCNA module genes and differentially expressed genes were considered the TMEIGs. **(G)** PCA plot demonstrates that the TMEIG subtypes display distinct gene expression patterns. **(H)** The survival analysis of TMEIG subtypes in the combined GEO cohort. **(I)** The boxplot of the immune score and stromal score between TMEIG subtypes. **(J)** The boxplot of seven immune checkpoint genes between TMEIG subtypes. **(K)** The distribution of 15 types of immune cells between TMEIG subtypes. **(L–N)** GSEA analysis of GO function, KEGG pathway, and Hallmark gene set between TMEIG subtypes. The difference between the two groups was assessed using the Wilcox test. The log-rank test was used for KM survival analysis.

To identify key gene modules in TME clusters 1 and 2, WGCNA analysis was employed. The WGCNA analysis processes are shown in Supplementary Figures S2A–E. Adaptive immunity and innate immunity were derived from ssGSEA analysis. Stromal score, immune score, estimate score, tumor purity, adaptive immunity, innate immunity, and TME clusters 1 and 2 were used as traits. The heat map of module–trait relationships is shown in Figure 4A. Results indicated that blue (cor = 0.79, p < 1e−200), brown (cor = 0.53, *p* = 2.2e−63), and green (cor = 0.98, p < 1e−200) modules displayed the high correlations with adaptive immunity (Figures 4B–D).

Thus, the blue, brown, and green modules were identified as the key modules. We performed GO and KEGG analyses to explore the biological functions of genes within the key modules. As shown in Supplementary Figures S2F–H, GO and KEGG terms were closely related to the immune function, such as regulation of immune system process, cytokine production, TNF signaling pathway, TNF superfamily cytokine production, and TNF superfamily cytokine production. In the three modules, 223 genes with GS > 0.2 and MM > 0.8 were identified as candidate genes. We used the “Limma” package to obtain the differentially expressed genes (DEGs) between TME clusters 1 and 2, and the results are shown in the volcano map (Figure 4E). *p*-value < 0.05 and logFC >0.5 were set as parameters, and 719 DEGs were obtained (698 upregulated and 21 downregulated). Since there were very few downregulated genes, we mainly used the upregulated genes to compare with the candidate genes of WGCNA. A total of 202 TMEIGs were eventually identified after comparing candidate genes with upregulated genes (Figure 4F).

### TMEIG Cluster a Has a Better Prognosis

A total of 202 TMEIGs were again used for unsupervised clustering in the combined GEO cohort, and the clustering process is shown in Supplementary Figures S3A–E. The clustering result was most stable when *k* = 2. PCA plot also revealed significant differences between the TMEIG subtypes (Figure 4G; Supplementary Table S7). The KM plots revealed that patients in TMEIG cluster A exhibited better OS (Figure 4H, log-rank test, *p* = 0.047). Similarly, the heat map of tumor-related pathways showed that cell cycle and DDR signatures were significantly decreased in TMEIG cluster B. Angio, transforming growth factor-beta (TGFβ), antigen processing machinery (APM), TGF-beta response signatures (TBRS) of fibroblasts (F-TBRS), and immune checkpoint signatures were increased in TMEIG cluster B as compared to that in cluster A (Supplementary Figure S3J). ESTIMATE analysis showed that TMEIG cluster B had higher immune and stromal scores than TMEIG cluster A (Figure 4I). CIBERSORT analysis showed that immunosuppressive cells (M0/M1/M2 macrophages) increased significantly in TMEIG cluster B, whereas immunoreactive cells (CD8 T cell, CD4 memory resting T cells, resting dendritic cells, and activated dendritic cells) decreased significantly compared to TMEIG cluster A (Figure 4K). GSEA results indicated that immune-related functions (activation of immune response, cytokine-cytokine receptor interaction, and inflammatory response) significantly varied between TMEIG cluster A and TMEIG cluster B (Figures 4L–N). These results demonstrated that TMEIG subtype clustering accurately reflected the differences between TME subtypes.

### Patients With High Tumor Microenvironment Immune Gene Scores Have a Poorer Prognosis in Multiple CRC Cohorts

**FIGURE 5:**
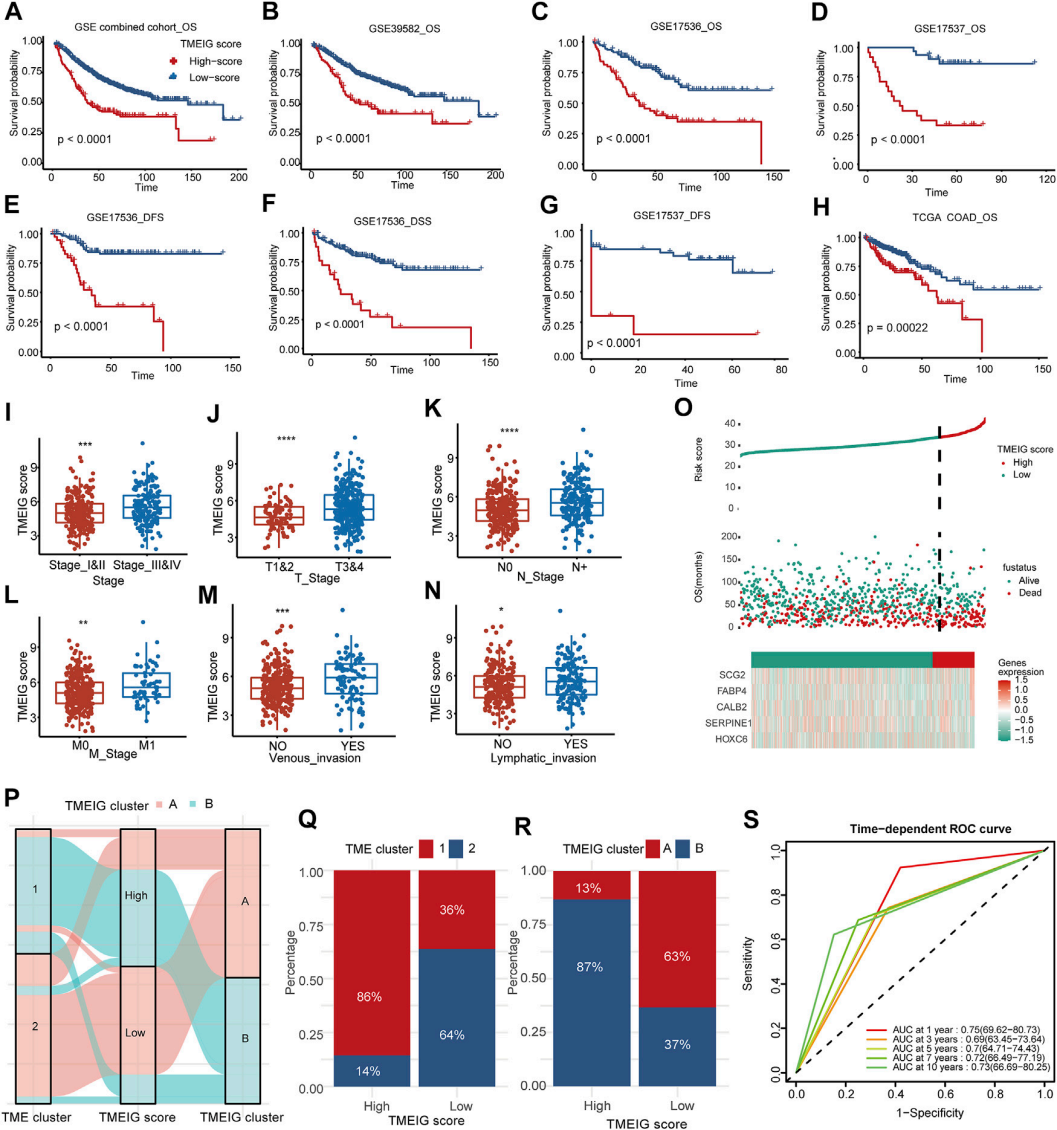
Clinical significance of TMEIG score. **(A–H)** The survival analysis of TMEIG score in multiple colorectal cancer cohorts. OS represents overall survival, DFS represents disease-free survival, and DSS represents disease-specific survival. **(I–N)** The relationship between clinicopathological parameters and TMEIG score in TCGA COAD. The TMEIG score was transformed to log2 format for analysis. Clinicopathological parameters are collated from the UCSC website. **(O)** The risk factor diagrams of the combined GEO cohort. **(P)** The Sankey diagram revealed the correlation between the TME cluster, TMEIG score, and TMEIG cluster in the combined GEO cohort. **(Q,R)** The stacked histogram exhibits the distribution of the TME cluster and TMEIG cluster between high and low TMEIG score groups. **(S)** ROC plot shows the predictive value of the TMEIG score combined with age, sex, M stage, and TNM stage in the GSE39582 cohort using stepwise Cox regression. The difference between the two groups was assessed using the Wilcox test. The log-rank test was used for KM survival analysis.

Gene signature is a simple and effective model widely used in clinical practice (Paik et al., 2004; van 't Veer et al., 2002; Parker et al., 2009). To further facilitate the application of TME subtypes in CRC, we intended to construct a TMEIG score system. First, 662 genes were obtained by comparing 2,799 genes in blue, brown, and green modules with 698 DEGs (Supplementary Figure S4A). Univariate Cox regression analysis was performed in the combined GEO cohort and TCGA COAD cohort. With a *p*-value less than 0.05, 287 and 47 prognostic genes were obtained from the combined GEO and TCGA COAD cohorts, respectively. Then, Lasso regression was used to identify 27 common genes in the combined GEO cohort (Supplementary Figure S4B). Details of the Lasso regression are shown in Supplementary Figures S4C and D. After cross-validating the results ten times, five genes (*SERPINE1*, *FABP4*, *SCG2*, *CALB2*, and *HOXC6*) and their corresponding lambda coefficients were obtained when lambda = 0.0713387182. The TMEIG score was constructed based on the expression and coefficient of the five genes as described in the methods. According to the optimal cutoff value, patients were divided based on whether their TMEIG scores were high or low (Supplementary Table S8). OS analysis suggested that the high TMEIG scores were associated with poor prognosis in patients with CRC (Figure 5A, log-rank test, *p* < 0.0001).

Internal validation cohorts indicated that the OS of the patients with high TMEIG scores was poorer than those with low scores (Figures 5B–D, GSE39582, GSE17536, GSE17537, log-rank test, *p* < 0.0001). In addition, the PFS and DSS in the low TMEIG score group were superior to those of the high TMEIG score group (Figures 5E–G, GSE17536 DFS, GSE17536 DSS, GSE17537 DFS, log-rank test, *p* < 0.0001). Then, the TCGA COAD cohort also revealed that the overall survival of the high TMEIG score group was poorer (Figure 5H; Supplementary Table S8). When analyzing the relationship between clinicopathological parameters and the TMEIG score, we observed that the scores of patients exhibiting stage III & IV, T 3 & 4, lymphatic invasion, and venous invasion were significantly higher (Figures 5I–N; Supplementary Table S9), suggesting the high TMEIG score was associated with poor clinical prognosis. Furthermore, the risk factor diagrams of the combined GEO and TCGA COAD cohorts indicated that the high TMEIG score group had significantly higher mortality than the low TMEIG score group (Figure 5O; Supplementary Figure S4E). All the results demonstrated poor prognoses in patients with high scores. The distribution of patients in the TME clusters, TMEIG clusters, and TMEIG score groups are shown in Figure 5P, which indicates that most patients in TME cluster 1 belonged to TMEIG cluster B and the high TMEIG score group. Consistent with the results, the high TMEIG score group had a higher proportion of patients in TME cluster 1 and TMEIG cluster B (Figures 5Q,R). Moreover, patients in TME cluster 1 and TMEIG cluster B exhibited higher TMEIG scores than that in TME cluster 2 and TMEIG cluster A (Supplementary Figures S4F,G). This evidence demonstrated that the TMEIG score could effectively surrogate TME and TMEIG subtypes. Furthermore, TMEIG score, age, sex, and T, N, M, and TNM stages were included in stepwise Cox regression in the GSE39582 cohort, which possessed comprehensive clinical information. Results indicated that TMEIG score combined with age, sex, M stage, and TNM stage exhibited the best predictive power (Supplementary Figure 5S, AUC: 0.75, 0.69, 0.7, 0.72, and 0.73 at 1, 3, 5, 7, and 10 years, respectively), and was validated in the TCGA COAD cohort (Supplementary Figure S4H).

### Patients With High Tumor Microenvironment Immune Scores Are More Likely to Benefit From Immune Checkpoint Blockers

**FIGURE 6:**
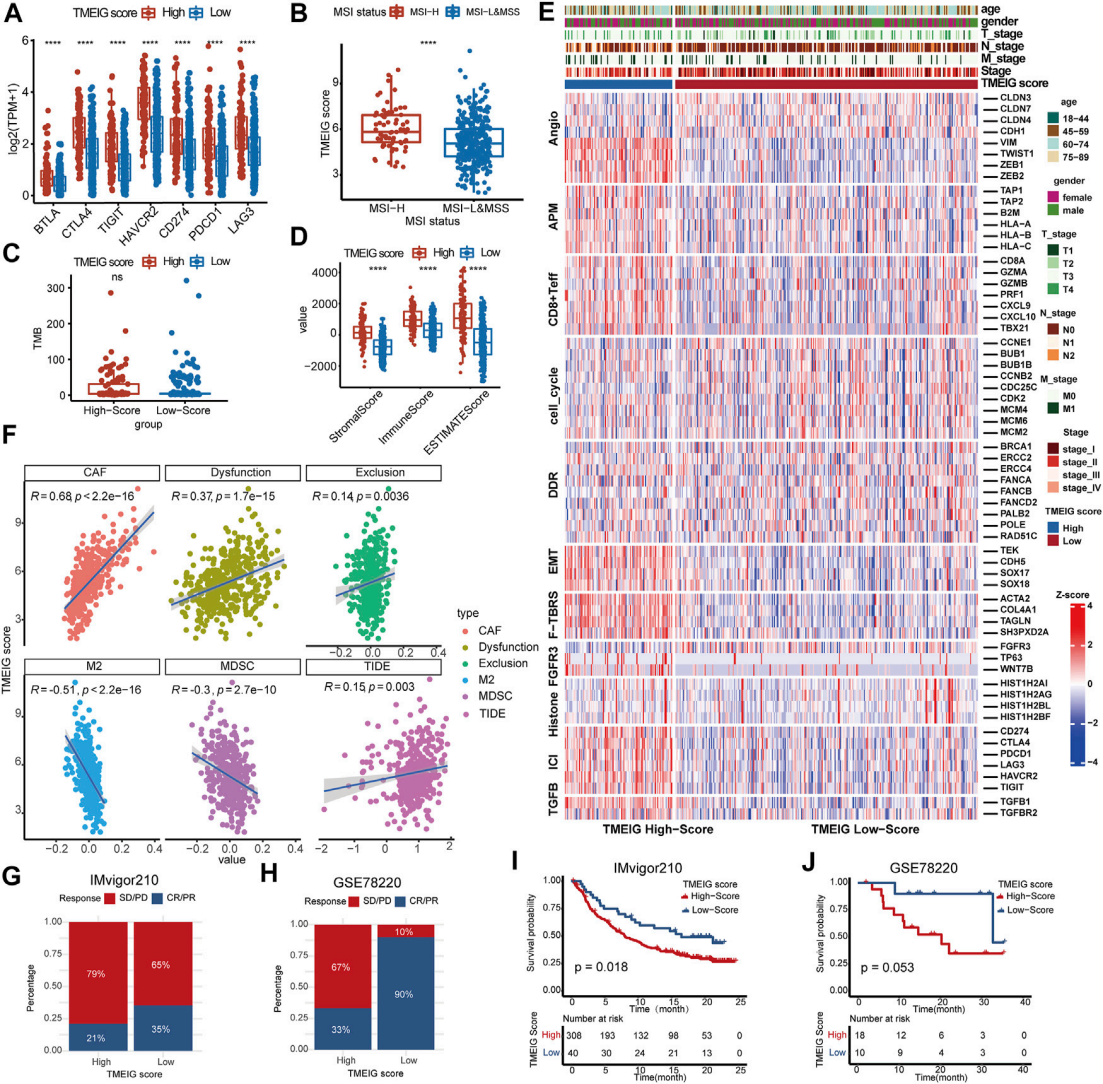
The correlation between TMEIG score and ICB response. **(A)** The boxplot of seven immune checkpoint genes between the high and low TMEIG score groups in the TCGA COAD cohort. **(B)** The TMEIG score between MSI-H and MSI-L/MSS subgroups. The boxplot showed that patients with MSI-H possessed higher TMEIG scores than MSI-L/MSS (Wilcox test, *p* = 6.9e−06). The TMEIG score was transformed to log2 format for analysis. **(C)** TMB difference in the high and low TMEIG score groups in the TCGA COAD cohort. **(D)** Relationship between TME subtypes and 11 critical biological pathways in the TCGA COAD cohort. Rows of the heat map represent gene expression grouped by pathway. **(F)** The Pearson correlation analysis between TMEIG score and tumor-associated fibroblast (CAF), T cell dysfunction (Dysfunction), T cell exclusion (Exclusion), M2 macrophage (M2), myeloid-derived suppressor cell (MDSCs), and TIDE score. The TMEIG score was transformed to log2 format for analysis. **(G,H)** The stacked histogram exhibits the distribution of ICB response rates between high and low TMEIG score groups in IMvigor210 and GSE78220 cohorts. The blue (CR/PR) indicates patients who responded to ICB, whereas the red (SD/PD) represents patients who did not respond to ICB treatment. **(I** and **J)** Survival analysis in ICB treatment cohorts (IMvigor210 and GSE78220) using the log-rank test. The difference between the two groups was assessed using the Wilcox test.

To evaluate whether the TMEIG score could predict the efficacy of ICB treatment, we analyzed the expression of crucial immune checkpoint molecules between high and low TMEIG score groups. The results showed that the expression of immune checkpoint molecules (*PD-1* [*PDCD1*], *PD-L1* [*CD274*], cytotoxic T-lymphocyte associated protein 4 [*CTLA4*], B- and T-lymphocyte attenuator [*BTLA*], T cell immunoglobulin and ITIM domain [*TIGIT*], hepatitis A virus cellular receptor 2 [*HAVCR2*], and lymphocyte-activation gene 3 [*LAG3*]) was significantly higher in the high score group (Figure 6A). Patients with CRC who have microsatellite instability-high (MSI-H) tumors are more likely to benefit from immune checkpoint inhibitors than patients with microsatellite stable (MSS)/MSI-low (MSI-L). To explore the relationship between TMEIG score and MSI status, the MSI status of TCGA COAD patients was downloaded from the supplements of previous studies focusing on MSI detection (Supplementary Table S10). There were 72 patients identified as MSI-H and 355 identified as MSI-L/MSS in TCGA COAD determined by MSI-PCR. We then analyzed whether the TMEIG score had prognostic value across MSI-H and MSI-L/MSS subgroups. KM plots demonstrated that patients with high TMEIG scores exhibited poor overall survival in MSI-H and MSI-L/MSS subgroups (Supplementary Figures S5A,B). Further analysis showed that patients with MSI-H possessed higher TMEIG scores (Figure 6B, *p* = 6.9e−06). Next, we explored the proportion of patients with MSI-H and MSI-L/MSS in high and low TMEIG score groups. We observed more MSI-H CRC patients in the high TMEIG score group (Supplementary Figure S5C, High: 26%, Low: 14%). Previous studies reported that TMB was a predictor of the efficacy of ICB therapy. When exploring the TMB of patients from the TCGA COAD cohort, results indicated no statistical difference between high and low TMEIG score groups (Figure 6C). In addition, the top six driver genes with the highest alteration frequency were further analyzed. The alteration frequency of *APC*, *TP53*, *TTN*, *KRAS*, *PIK3CA*, and *MUC16* in high and low TMEIG score groups are displayed in Supplementary Figure S5D. The ESTIMATE algorithm revealed that immune, stromal, and estimate scores were significantly higher in the high TMEIG score group (Figure 6D). Furthermore, pathway heat maps demonstrated that EMT, angiogenesis (vimentin [*VIM*], Twist-related protein 1 [*TWIST1*], zinc finger E-box binding homeobox 1 and 2 [*ZEB1* and *ZEB2*]), and T-cell factor-beta (TCFβ) signatures were significantly more activated in the high TMEIG score group. In contrast, cell cycle and DDR signatures were highly expressed in the low TMEIG score group (Figure 6E). These results indicated significant differences in the TME and biological pathways between high and low TMEIG score groups. To dissect the relationship between TMEIG score and ICB response, we used the TIDE algorithm to predict ICB response based on transcriptome signatures. The TIDE algorithm (Figure 6F) showed that the TMEIG score was positively correlated with CAF (R = 0.68, *p* < 2.2e−16), Dysfunction (R = 0.37, *p* < 1.7e−15), and Exclusion (R = 0.14, *p* < 0.0036), and negatively correlated with M2 macrophages (R = 0.51, *p* < 2.2e−16) and MDSC (R = −0.3, *p* < 2.7e−10). In addition, there was a strong positive correlation between the TMEIG and TIDE scores (Figure 6F, R = −0.15, *p* < 0.003).

Then, we explored the predictive power of the TMEIG score in two ICB therapy cohorts. In IMvigor210 and GSE78220 cohorts, the ICB response rates were significantly lower in the high TMEIG score group (Figures 6G,H). Notably, patients with high TMEIG scores exhibited worse overall survival (Figures 6I,J; IMvigor210: log-rank test, *p* = 0.018, GSE78220: log-rank test, *p* = 0.053). The insignificant result in GSE78220 (28 patients) can be attributed to the small sample size. Furthermore, the biomarker evaluation module on the TIDE website was used to assess the accuracy of the TMEIG score using multiple ICB cohorts as compared to other published biomarkers. The TMEIG score demonstrated an AUC of more than 0.5 in nine out of 16 ICB cohorts (Supplementary Figure S6), demonstrating its robustness as a predictive biomarker (Fu et al., 2020).

### The Biomarker Genes Are Differentially Expressed in CRC and Significantly Correlate With Immune Cells

**FIGURE 7:**
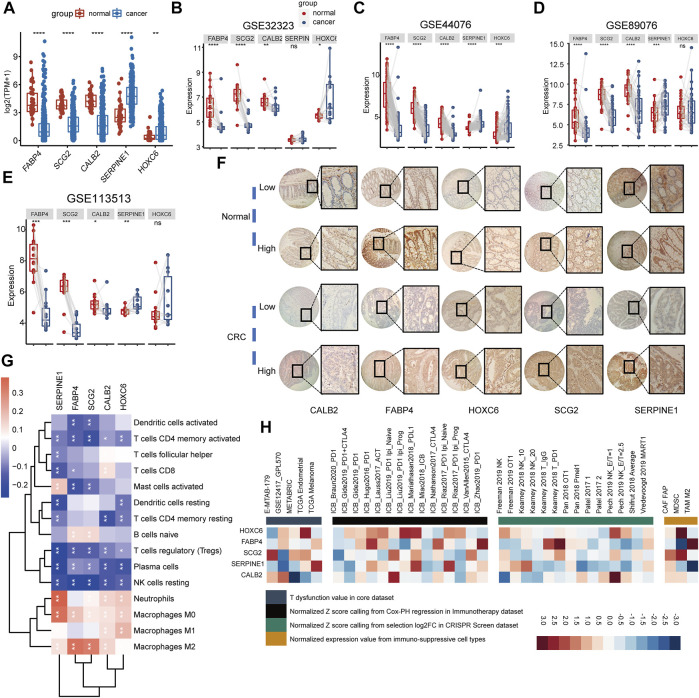
Exploring the biological functions of biomarker genes. **(A)** Comparison of biomarker gene expression between normal tissue and cancer tissue in TCGA COAD. **(B–E)** Comparison of biomarker gene expression between cancer tissues and paired normal tissues, statistically assessed using Wilcox test. **(F)** The representative immunohistochemical images of FABP4, SCG2, CALB2, SERPINE1, and HOXC6. A total of 16 pairs of CRC tissue (cancer and adjacent normal tissue) were collected for IHC. **(G)** The heatmap shows the Pearson’s correlation between five biomarker genes and immune cells in the combined GEO cohort. Red represents positive correlation, whereas blue represents negative correlation. **(H)** The correlation between five biomarker genes and four immunosuppressive indices (columns), including T cell dysfunction score (first column, T dysfunction value in core dataset), association with ICB survival outcome (second column, z-score in the Cox-PH regression in immunotherapy), log-fold change (logFC) in CRISPR screens (third column, helping identify regulators whose knockout can mediate the efficacy of lymphocyte-mediated tumor killing in cancer models), and T cell exclusion score (fourth column, assessing the gene expression levels in immunosuppressive cell types that drive T cell exclusion). Genes (rows) are ranked by average value across four immunosuppressive indices analyzed using the TIDE website.

To further understand the functions of the biomarker genes consisting of the TMEIG score, we analyzed the expression levels of *SERPINE1*, *FABP4*, *SCG2*, *CALB2*, and *HOXC6* in the TCGA-COAD cohort. The results demonstrated that the expression values of *SERPINE1* and *HOXC6* were significantly upregulated in tumors, whereas *FABP4*, *SCG2*, and *CALB2* were highly expressed in normal tissues (Figure 7A). Furthermore, the same results were found in paired differential expression in multiple CRC cohorts (Figures 7B–E, GSE32323, GSE44076, GSE89076, and GSE113513). Our immunohistochemistry (IHC) results revealed that *CALB2* exhibited relevant stronger staining in two cases of tumor (2/16), with the other 14 cases displaying low expression (14/16). For *FABP4*, seven and two cases in tumor and normal samples, respectively, exhibited stronger staining, whereas nine and 14 cases in tumor and normal samples, respectively, were with low staining intensity. For *HOXC6*, seven and nine samples out of 16 exhibited strong staining in normal and tumor samples, respectively. The protein level of *SCG2* was high in 12 cases of CRC samples (12/16) and 11 cases of normal samples (11/16). *SEP1NG1* was found strongly stained in 11 cases of tumor samples (11/16) and nine cases of normal samples (9/16). Representative immunohistochemical images are shown in Figure 7F, and the high-resolution images are shown in Supplementary Figure S7. The qPCR experiments (Supplementary Figure S8A) revealed that the expression of *HOXC6* and *SERPINE1* was significantly upregulated in RKO and HT29 cell lines, and *FABP4* expression was downregulated in nearly all CRC cell lines analyzed. Although *SCG2* and *CALB2* were downregulated in patients with CRC in multiple cohorts, qPCR experiments showed that their expression was upregulated in several CRC cell lines (such as HCT116 and HT29). This discrepancy may be due to false positives in RNA sequencing or the heterogeneity between clinical tissues and tumor cells. Studies involving more clinical samples or cell lines may be needed to confirm the expression of the two genes at the RNA and protein levels in the future. KM plots of these genes are displayed in Supplementary Figure S9A. Results indicated that all genes were closely related to OS. GSEA analysis indicated that the five genes were involved in multiple cancer biological functions: cell motility, angiogenesis, cell migration, programmed cell death, MAPK signaling pathway, and PI3K-Akt signaling pathway. Notably, the immune-related pathway “cytokine-cytokine receptor interaction” was also significantly enriched in most of these genes (*SERPINE1*, *FABP4*, *SCG2*, and *HOXC6*) (Supplementary Figure S9B). Next, we summarized several immunological molecules from our previous studies, such as immune checkpoint genes and cytotoxic genes, and analyzed the correlation with five genes in TCGA COAD (Supplementary Figures S8B,C). Results demonstrated that most of the five genes were significantly correlated with immune checkpoint genes (*BTLA*, *CD274*, *CTLA4*, *HAVCR2*, *LAG3*, *PDCD1*, and *TIGIT*) and cytotoxic genes (granzyme A [*GZMA*], *GZMB*, *GZMK*, *GZMM*, interferon-gamma [*IFNG*], perforin 1 [*PRF1*], and tumor necrosis factor superfamily member 11 [*TNFSF11*]), which revealed that these genes might play an important role in tumor immunity. We then explored the correlation between the five genes and immune cells infiltrating the TME. As shown in the correlation heatmap, the five genes were positively related to macrophages (such as M0, M1, and M2), inversely correlated with resting NK cells and resting memory CD4^+^ T cells, CD8^+^ T cells, and activated memory CD4^+^ T cells (Figure 7G), which might explain the poor ICB response in the high TMEIG score group. Moreover, the gene set prioritization module on the TIDE website indicated that *HOXC6* was the most appropriate target to treat TME resistance to ICB (Figure 7H). The expression of *HOXC6* was positively associated with T cell dysfunction in GSE12417, METABRIC, and TCGA Endometria datasets (Figure 7H, left panel). In addition, high *HOXC6* expression was also associated with poorer ICB outcomes in multiple cohorts treated with ICB (Figure 7H, second to left panel). Among the immune-suppressive cell types, *HOXC6* was highly expressed on the MDSC and CAF (Figure 7H, right panel).

## Discussion

Understanding the heterogeneity of the tumor microenvironment is required to elucidate the biological properties of CRC and guide the treatment strategies. Moreover, the TME heterogeneity is closely related to the efficacy of ICB therapy (Lee et al., 2014; Nishino et al., 2017; Cristescu et al., 2018; Mariathasan et al., 2018). Thus, understanding TME heterogeneity may provide new insights into CRC immunotherapy.

In this study, we constructed TME subtypes based on the TME landscape of patients with CRC, which can accurately distinguish the heterogeneity of the TME and predict the clinical prognosis. The patients were then re-clustered by TMEIGs identified by WGCNA and differential expression analysis. Two TMEIG subtypes were obtained, reflecting heterogeneity in TME and clinical prognosis. Gene signature is a simple and effective model widely used in clinical practice (Paik et al., 2004; van 't Veer et al., 2002; Parker et al., 2009). Therefore, we established a TMEIG score system to quantify the TME heterogeneity in patients with CRC. The Sankey plots revealed that the TME and TMEIG subtypes were consistent with the TMEIG score, suggesting that the TMEIG score could be utilized as a surrogate biomarker of TME heterogeneity.

Tumor mutation burden (TMB) (Chan et al., 2019), microsatellite instability (MSI) status (Ganesh et al., 2019), and immune checkpoint genes are important factors affecting ICB therapy. Patients with high levels of TMB and MSI-H exhibited better ICB therapy responses. In this study, there was no significant difference in TMB between high and low TMEIG score groups. However, patients with MSI-H possessed a higher TMEIG score, and there were more patients with MSI-high CRC in the high TMEIG score group. In addition, the expression of immune checkpoint molecules was higher in patients with high TMEIG scores. Patients with high expression of PD-L1 and PD-1 are more likely to benefit from ICB therapy (Topalian et al., 2012; Nishino et al., 2017). These results indicate that patients with high TMEIG scores tend to respond better to ICB therapy. However, ICB response is influenced by numerous factors, such as EMT (Jiang and Zhan, 2020), angiogenesis (Tian et al., 2017; Voron et al., 2014), and the TCF-β pathway (Mariathasan et al., 2018; Tauriello et al., 2018). EMT, angiogenesis, and TCFβ pathway activation inhibit the efficacy of immune checkpoint therapy. The pathway heatmap revealed that EMT, angiogenesis (*VIM, TWIST1, ZEB1*, and *ZEB2*), and TCFβ signatures were significantly activated in the high TMEIG score group (Figure 7E). Furthermore, the TIDE score predicted the efficacy of ICB therapy based on two mechanisms of tumor immune escape (T cell dysfunction and T cell exclusion) (Jiang et al., 2018). A higher TIDE score is related to poorer ICB response and survival in patients receiving anti–PD-1 and anti-CTLA4 therapies (Jiang et al., 2018). In the present study, the TMEIG score was positively related to dysfunction and exclusion scores (Figure 6F). It indicated that patients with high TMEIG scores possessed fewer CTL cells, which were majorly dysfunctional in the TME. In line with the above results, the TMEIG score was positively correlated with the TIDE score (R = 0.15, *p* = 0.003), indicating that patients in the high TMEIG score group exhibited poorer ICB response. The prediction of ICB response by MSI, TMB, or PD-L1 is based on the presence of CTL cells in the TME. Hence, we speculated that patients with high TMEIG scores mainly tend to exhibit poor ICB therapy response due to fewer CTL cells that are primarily dysfunctional. Since there was no suitable public ICB treatment CRC cohorts at the time of publication, we only used transcriptome data from other tumor types to verify the predictive power of the TMEIG score. Nevertheless, validation in melanoma and urothelial cancer datasets may indirectly suggest that the TMEIG score predicts the efficacy of immune checkpoint therapy in CRC. In accordance with TIDE results, a higher TMEIG score was associated with poorer ICB response and prognosis in two ICB treatment cohorts. In conclusion, the evidence demonstrated that the TMEIG score might serve as a reliable ICB biomarker in CRC. We will further validate our results once transcriptome data of CRC patients undergoing immune checkpoint therapy becomes publicly available or establish our own cohort regarding this point.

In our study, the TMEIG score was determined by *SERPINE1, FABP4, SCG2, CALB2*, and *HOXC6* expression. *HOXC6* is a member of the homeobox family, which encode transcription factors that play a critical role in morphogenesis in all multicellular organisms. *HOXC6* expression was higher and negatively associated with prognosis in right-sided colon cancer than in left-sided colon cancer. This finding was further validated by tissue microarray analysis. *HOXC6* facilitated proliferation and metastasis through the dickkopf-1 (*DKK1*)/Wnt/β-catenin axis in right-sided colon cancer (Qi et al., 2021; Garris et al., 2018). The role of *FABP4*, which encodes the fatty acid–binding protein found in adipocytes, is unclear in CRC. A study demonstrated that *FABP4* was downregulated in CRC (Zhao et al., 2019). IHC and ELISA data from another study revealed that *FABP4* and plasma FABP4 concentrations were higher in CRC tissues than in normal tissues (Zhang et al., 2021). Thus, the role of *FABP4* in CRC must be investigated further. In addition, mRNA and protein levels of *SCG2*, a member of the chromogranin family of acidic secretory proteins, were significantly downregulated in CRC tissues (Wang et al., 2021; Fang et al., 2021). Mechanistically, *SCG2* inhibits tumor growth and angiogenesis by disrupting the activities of HIF-1α/VEGF in malignant CRC tissues (Fang et al., 2021). *In vitro* and *in vivo* studies have shown that *CALB2* promotes hepatocellular carcinoma metastasis *via* the TRPV2-Ca2+-ERK1/2 signaling pathway (Chu et al., 2022). Although fluorouracil (5-FU) treatment reduced the mRNA and protein expression of *CALB2* in CRC, their expression levels were not quantified and compared in tumor and normal tissues (Stevenson et al., 2011). *SERPINE1* expression is reportedly upregulated in CRC tissues and is associated with tumor invasiveness and aggressiveness (Mazzoccoli et al., 2012). Our study also reports the same trend (Figures 7A–E). Nevertheless, the roles of *HOXC6*, *SERPINE1*, *FABP4*, *SCG2*, and *CALB2* in tumorigenesis, cancer immunity, and ICB treatment are poorly understood. In the present study, IHC and qPCR results preliminarily elucidated the expression levels of these five molecules in CRC and normal tissues. Larger clinical sample sizes are required to verify mRNA and protein expression levels reported in this study and whether protein levels can be used to predict the prognosis of patients suffering from CRC and their response to ICB therapy. In addition, we observed that the five genes were significantly associated with immune cells of TME, immune checkpoint genes, and cytotoxic genes. Immune checkpoint genes and cytotoxic genes were collected from our previous study (Wang et al., 2022). Moreover, heatmaps also demonstrated that these genes, especially *HOXC6*, were closely associated with four immunosuppressive indices, including T cell dysfunction score, T cell exclusion score, association with ICB survival outcome, and logFC in CRISPR screens. Collectively, the roles of the five genes in tumor immunity are worthy of investigation, which will be the focus of our future research.

Our study has numerous advantages. First, datasets of the combined GEO cohort were downloaded from the GPL570 platform, which reduced the batch effect caused by different platform processes. Second, a large cohort with more than 1,000 samples was used for clustering, guaranteeing stable clustering results. Third, the prognostic power and predictive ICB response of the TMEIG score have been validated in multiple cohorts. However, the study design does have a few drawbacks. First, the predictive ICB response power of the TMEIG score was assessed in melanomas and metastatic urothelial cancer. These data must be verified using patients with CRC. Second, the relationship between the five molecules of the TMEIG score system and tumorigenesis, immune system, and ICB response were not investigated in this study. Future *in vivo* and *in vitro* studies from our group will focus on these aspects.

In conclusion, we identified the TME subtypes that comprehensively depicted the TME, revealed multiple aspects of CRC biology, and assessed variation in the prognosis of patients with CRC. TMEIG score is a robust marker to predict patients’ prognosis and may serve as a predictor of ICB response in CRC. Moreover, we identified several potential targets that may play a critical role in ICB treatment, of which *HOXC6* may be the most significant.

## Data Availability

The original contributions presented in the study are included in the article/Supplementary Material; further inquiries can be directed to the corresponding authors.

## Supplementary Material

Supplementary Figure S1 Processes of constructing TME subtypes. **(A–D)** Consensus matrixes of the combined GEO cohort for each *k* (*k* = 2–5), displaying the clustering stability using 1000 iterations of hierarchical clustering. **(E)** Empirical cumulative distribution function plots display consensus distributions for each *k*. When *k* = 2, the distribution reaches an approximate maximum, indicating maximum stability.

Supplementary Figure S2 Details of the WGCNA analysis. **(A** and **B)** Analysis of the scale-free fit index and the mean connectivity for various soft-thresholding power values. **(C)** Hierarchical clustering dendrograms of co-expressed genes in modules. **(D** and **E)** The correlation between modules. **(F–H)** The GO and KEGG enrichment terms are in blue, brown, and green modules.

Supplementary Figure S3 Details of constructing TMEIG subtypes. **(A–D)** Consensus matrixes of the combined GEO cohort for each *k* (*k* = 2–5), displaying the clustering stability using 1000 iterations of hierarchical clustering. **(E)** Empirical cumulative distribution function plots display consensus distributions for each *k*. When *k* = 2, the distribution reaches an approximate maximum, indicating maximum stability. **(F)** Relationship between TMEIG subtypes and 11 critical biological pathways.

Supplementary Figure S4 Details of constructing the TMEIG score system. **(A)** The Venn diagrams show the intersection between genes in blue, brown, and green modules and DEGs. **(B)** The intersection of prognostic genes in the TCGA COAD cohort and the combined GEO cohort. **(C,D)** Details of the Lasso regression. **(E)** The risk factor diagrams of the TCGA COAD cohort. **(F** and **G)** The TMEIG score between TME Clusters as well as TMEIG Cluster. **(H)** ROC plot shows the predictive value of the TMEIG score combined with age, sex, M stage, and TNM stage in the TCGA COAD cohort using stepwise Cox regression.

Supplementary Figure S5 Exploring the TMEIG score groups. **(A)** The KM plot of high and low TMEIG groups in MSI-H subgroups (log-rank *p-*value = 0.037). **(B)** The KM plot of high and low TMEIG groups in MSI-L/MSS subgroups (log-rank *p-*value = 0.0039). **(C)** The stacking histogram shows the proportion of patients with MSI-H and MSI-L/MSS in high and TMEIG score groups. Red represents patients with MSI-H, and blue represents those with MSI-L/MSS. **(D)** The OncoPrint shows the top six mutated genes between high and low TMEIG score groups, including *APC*, *TP53*, *TTN*, *KRAS*, *PIK3CA*, and *MUC16*. There are 100 and 274 patients in the high and low TMEIG score groups, respectively. Individual patients are represented in each column.

Supplementary Figure S6 Comparison of TMEIG score and other biomarkers. AUC is employed to evaluate the prediction performance of the TMEIG score (Custom) and other common biomarkers on ICB response in 16 ICB treatment cohorts using the TIDE website.

Supplementary Figure S7 High-definition images of IHC.

Supplementary Figure S8 Exploring the role of the five biomarker genes. **(A)** The qPCR data of *FABP4*, *SCG2*, *CALB2*, *SERPINE1*, and *HOXC6*. NCM460 is a normal human colonic epithelial cell line, whereas SW620, RKO, HCT116, and HT29 are human CRC cell lines. NS, not significant. The statistical significance was assessed using one-way ANOVA. **(B)** Pearson correlation between the five genes and immune checkpoint genes (*BTLA*, *CD274*, *CTLA4*, *HAVCR2*, *LAG3*, *PDCD1*, and *TIGIT*) in TCGA COAD. **(C)** Pearson correlation between the five genes and cytotoxic genes (*GZMA*, *GZMB*, *GZMK*, *GZMM*, *IFNG*, *PRF1*, and *TNFSF11*) in TCGA COAD.

Supplementary Figure S9 Survival and GSEA analyses of the five biomarker genes. **(A)** Survival analysis of *SERPINE1*, *FABP4*, *SCG2*, *CALB2*, and *HOXC6* in the TCGA COAD cohort. **(B)** GSEA analysis of *SERPINE1*, *FABP4*, *SCG2*, *CALB2*, and *HOXC6* in the combined GEO cohort. Data from “c2.cp.kegg.v6.2.symbols.gmt” and “c5.all.v7.0.symbols.gmt” in the MSigDB website were chosen as the reference gene sets.

Supplementary Table S1 Cohorts information used in this study.

Supplementary Table S2 Survival data of the combined GEO and TCGA COAD cohorts.

Supplementary Table S3 Details of immunohistochemistry experiment in this study.

Supplementary Table S4 CIBERSORT results of the combined GEO cohort.

Supplementary Table S5 TME clusters in the combined GEO cohort.

Supplementary Table S6 Results of GSEA analysis between TME clusters in the combined GEO cohort.

Supplementary Table S7 TMEIG clusters in the combined GEO cohort.

Supplementary Table S8 TMEIG score in the combined GEO and TCGA COAD cohorts.

Supplementary Table S9 Clinical data of the TCGA COAD cohort.

Supplementary Table S10 MSI information of the TCGA COAD cohort.
