# Supplementary material for: Tumor Microenvironment Heterogeneity-Based Score System Predicts Clinical Prognosis and Response to Immune Checkpoint Blockade in Multiple Colorectal Cancer Cohorts
Source: Front Mol Biosci. 2022 Jun 28;9:884839. doi: 10.3389/fmolb.2022.884839 (PMC9274205; doi:10.3389/fmolb.2022.884839)
Supplement: Supplementary file 3 [file Table1.docx]

| Dataset | Experiment type | Company/Platform | | Sample | Usage |
| --- | --- | --- | --- | --- | --- |
| GSE17536 | array | Affymetrix-GPL570 | 177 | | GEO combined cobort |
| GSE17537 | array | Affymetrix-GPL570 | 55 | | GEO combined cobort |
| GSE14333 | array | Affymetrix-GPL570 | 29 | | GEO combined cobort |
| GSE39582 | array | Affymetrix-GPL570 | 58 | | GEO combined cobort |
| GSE72968 | array | Affymetrix-GPL570 | 68 | | GEO combined cobort |
| TCGA-COAD | sequencing | Illumina | 571 | | Survival analysis  Differential expression analysis |
| GSE44076 | array | Affymetrix- GPL13667 | 246 | | Paired differential expression analysis (196 samples) |
| GSE32323 | array | Affymetrix- GPL570 | 44 | | Paired differential expression analysis (34 samples) |
| GSE89076 | array | Agilent- GPL16699 | 80 | | Paired differential expression analysis (74 samples) |
| GSE113513 | array | Affymetrix- GPL15207 | 28 | | Paired differential expression analysis (28 samples) |
