# Supplementary figures and images for: Tumor Microenvironment Heterogeneity-Based Score System Predicts Clinical Prognosis and Response to Immune Checkpoint Blockade in Multiple Colorectal Cancer Cohorts
Source: Front Mol Biosci. 2022 Jun 28;9:884839. doi: 10.3389/fmolb.2022.884839 (PMC9274205; doi:10.3389/fmolb.2022.884839)

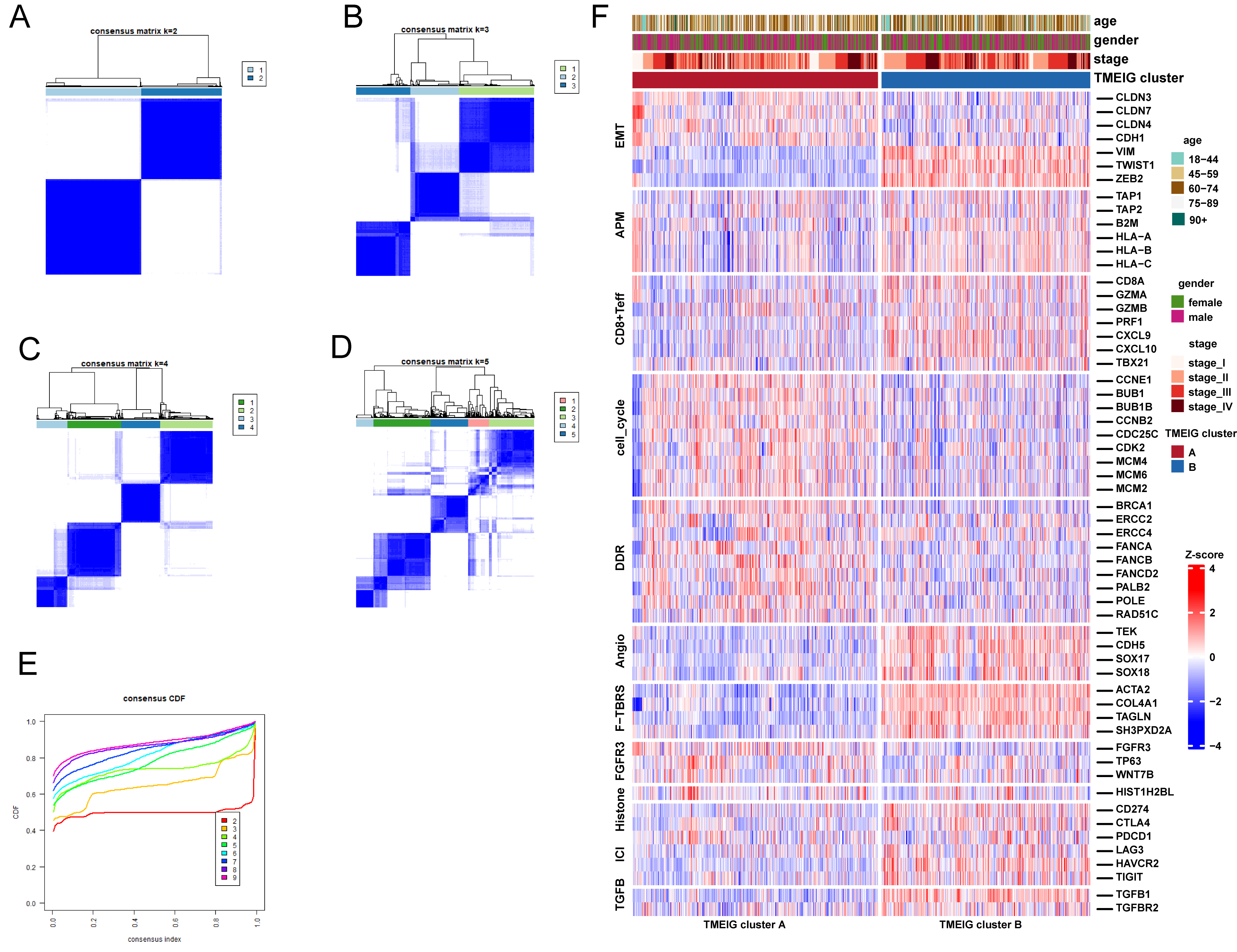

Supplement: Supplementary file 4 [file Image3.tif]

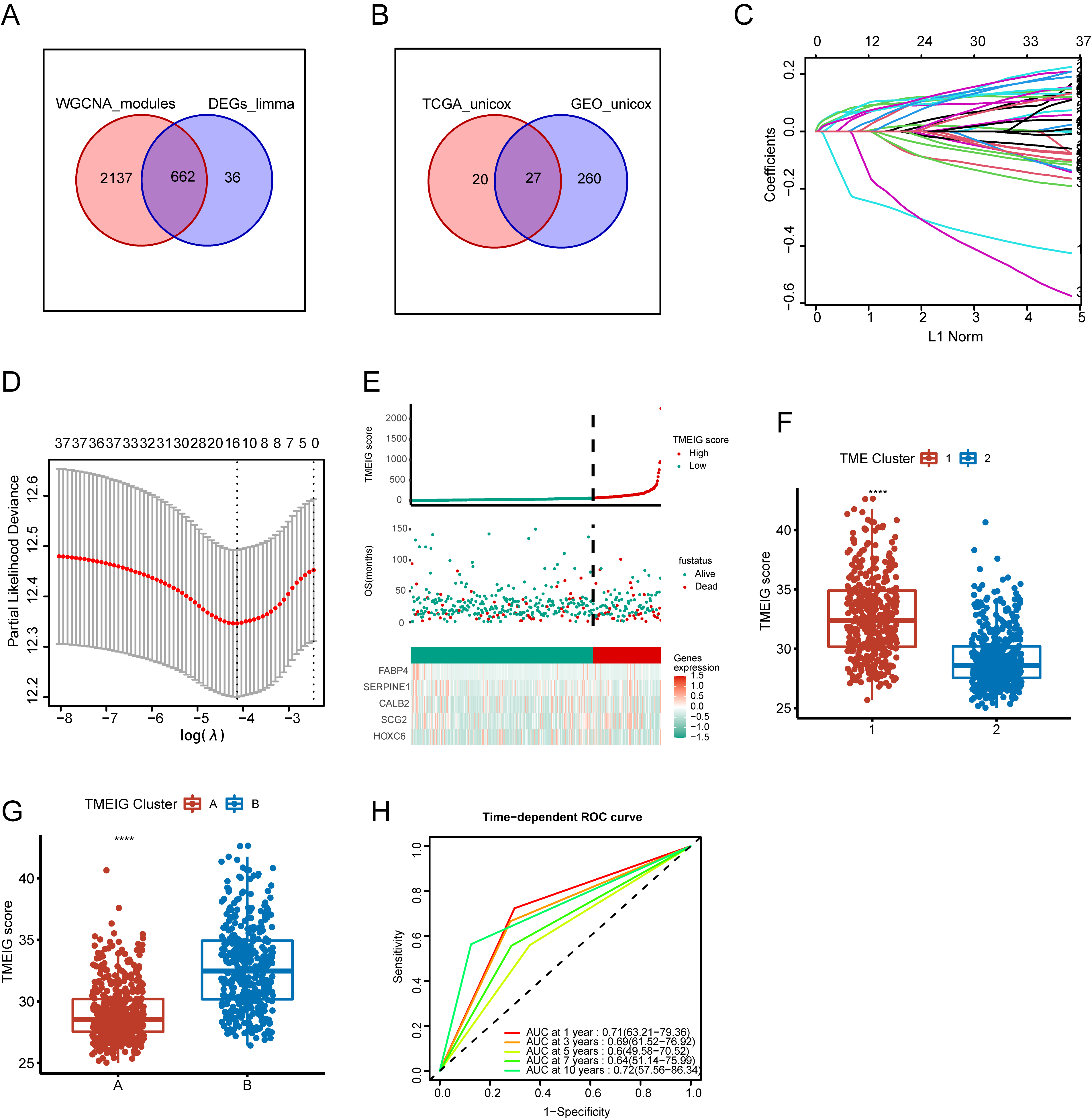

Supplement: Supplementary file 5 [file Image4.tif]

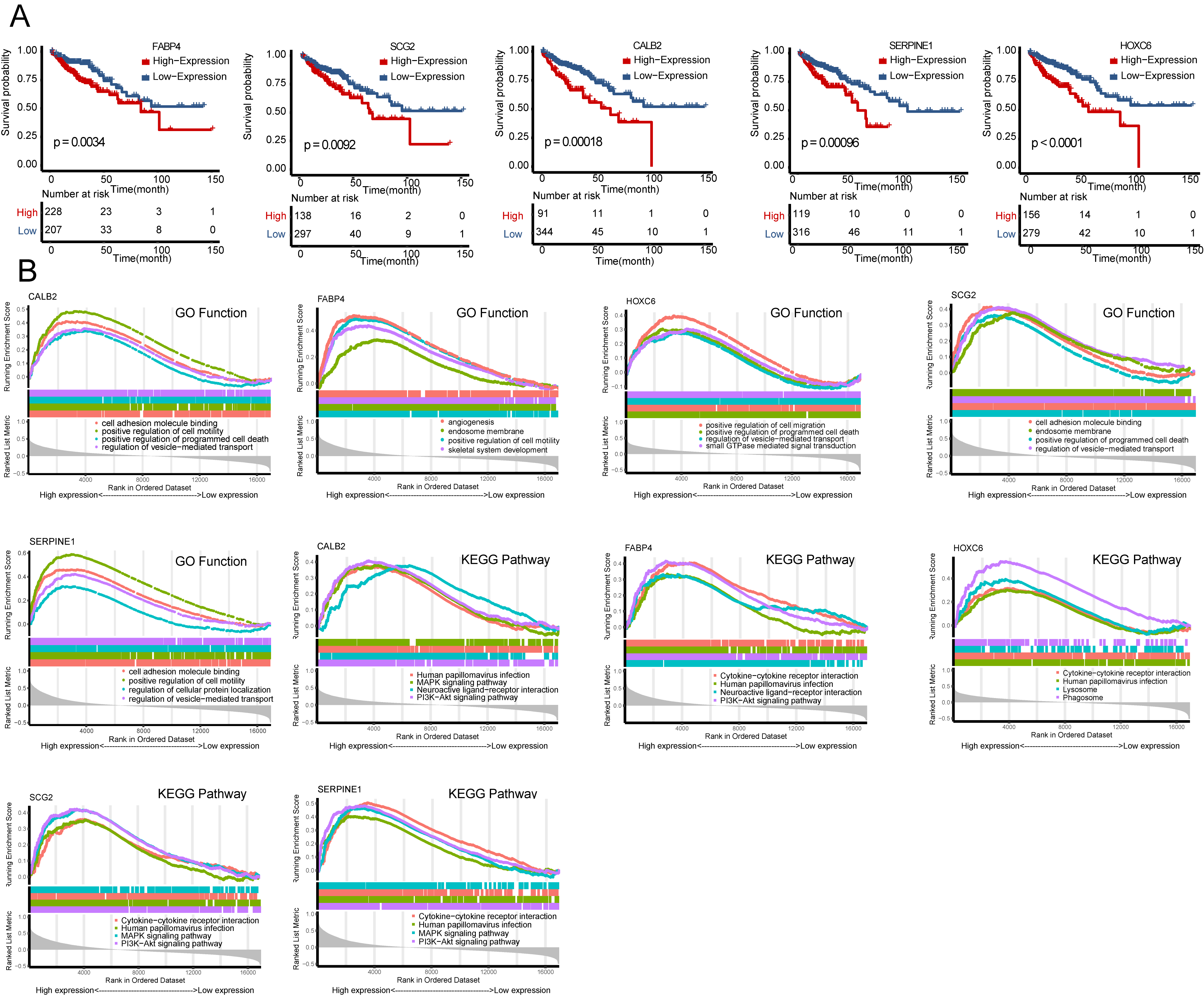

Supplement: Supplementary file 6 [file Image9.tif]

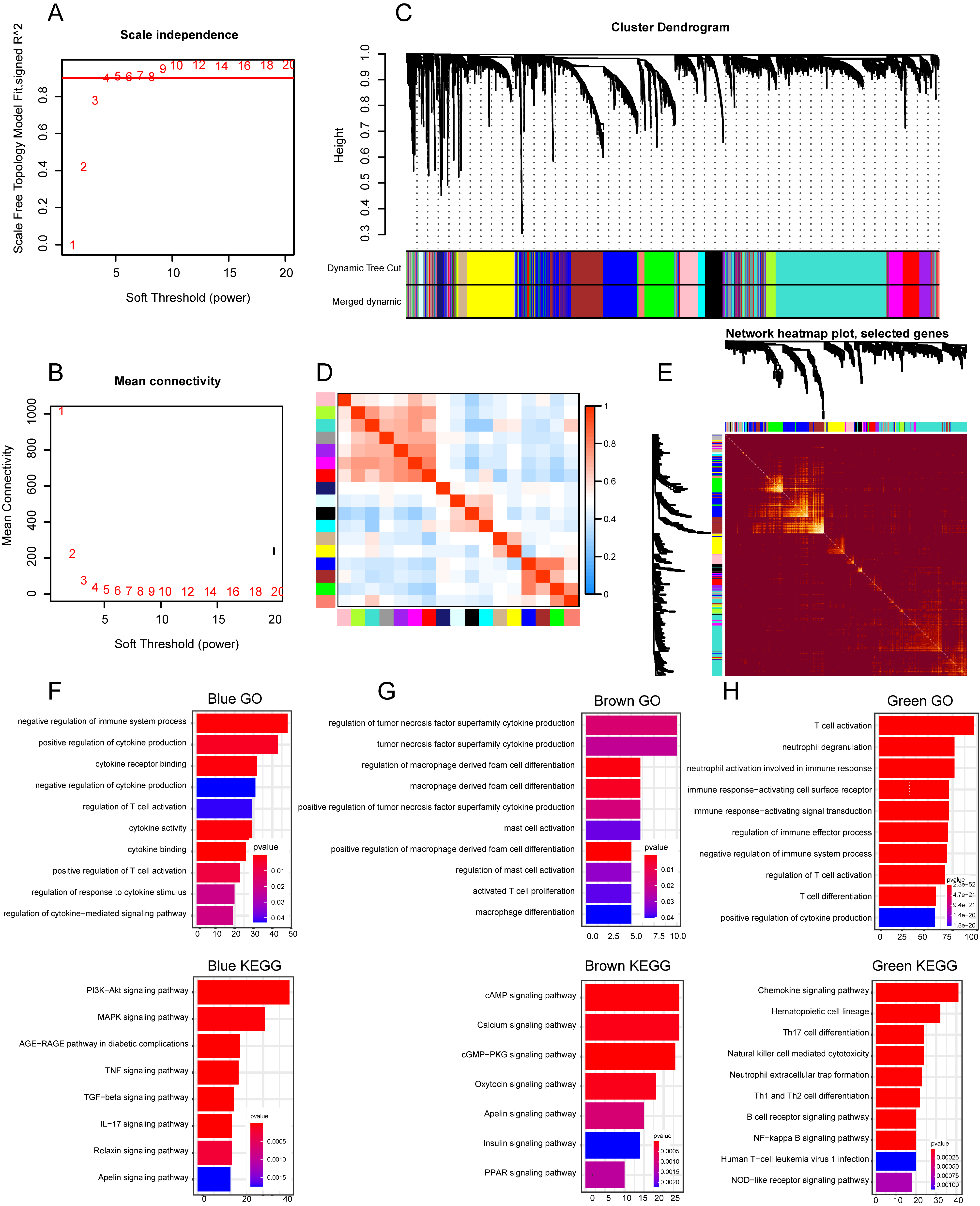

Supplement: Supplementary file 7 [file Image2.tif]

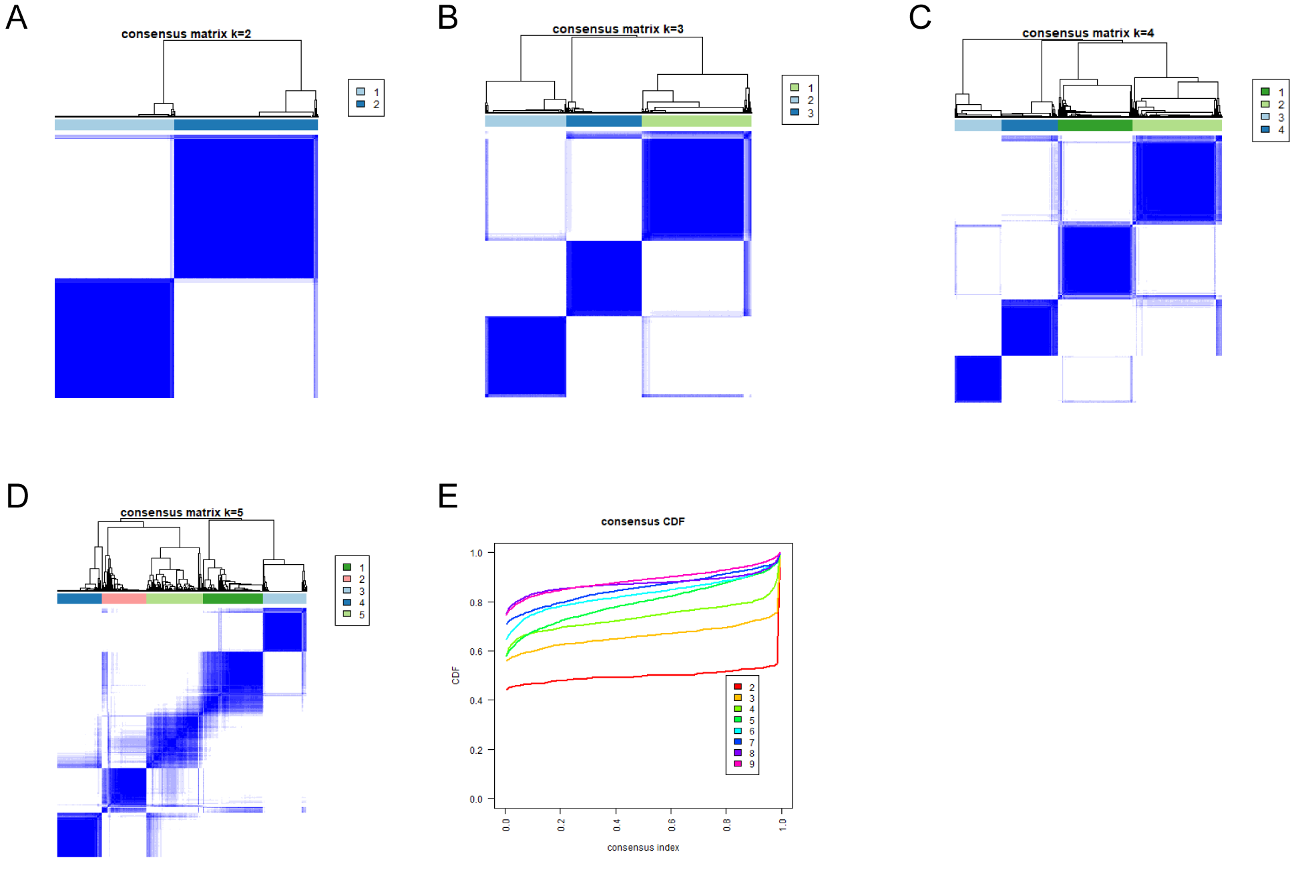

Supplement: Supplementary file 10 [file Image1.tif]

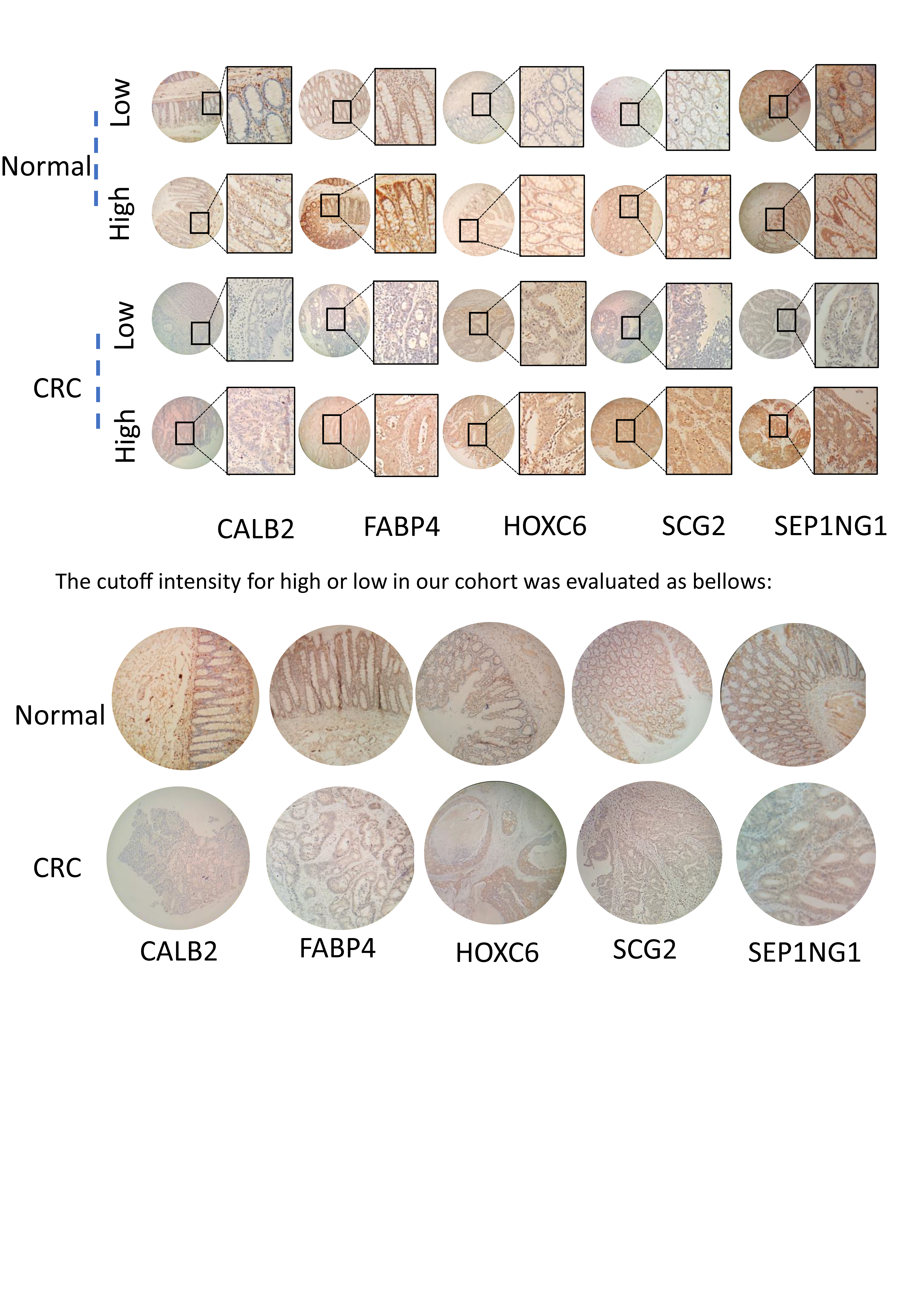

Supplement: Supplementary file 11 [file Image7.TIF]

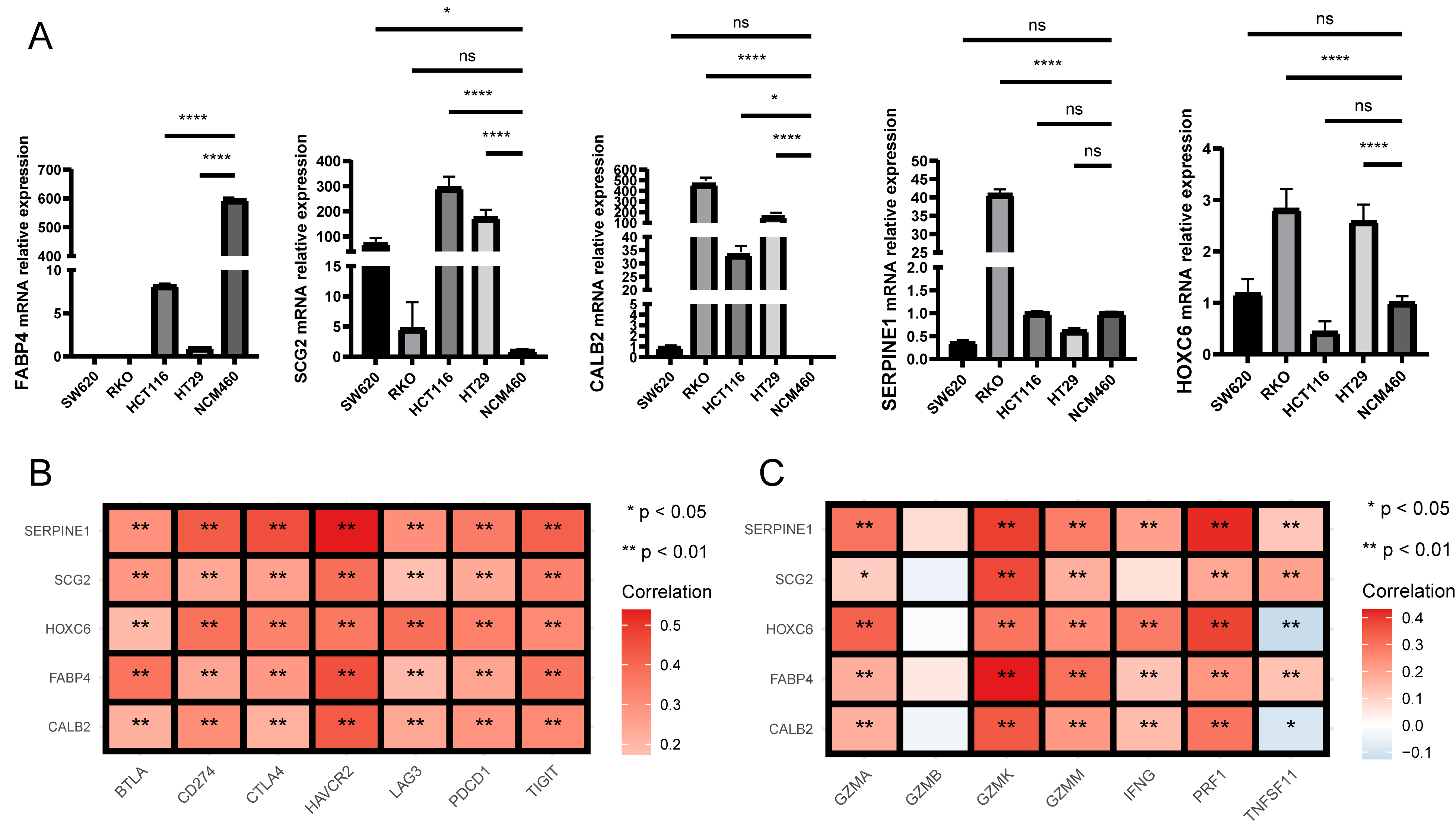

Supplement: Supplementary file 14 [file Image8.tif]

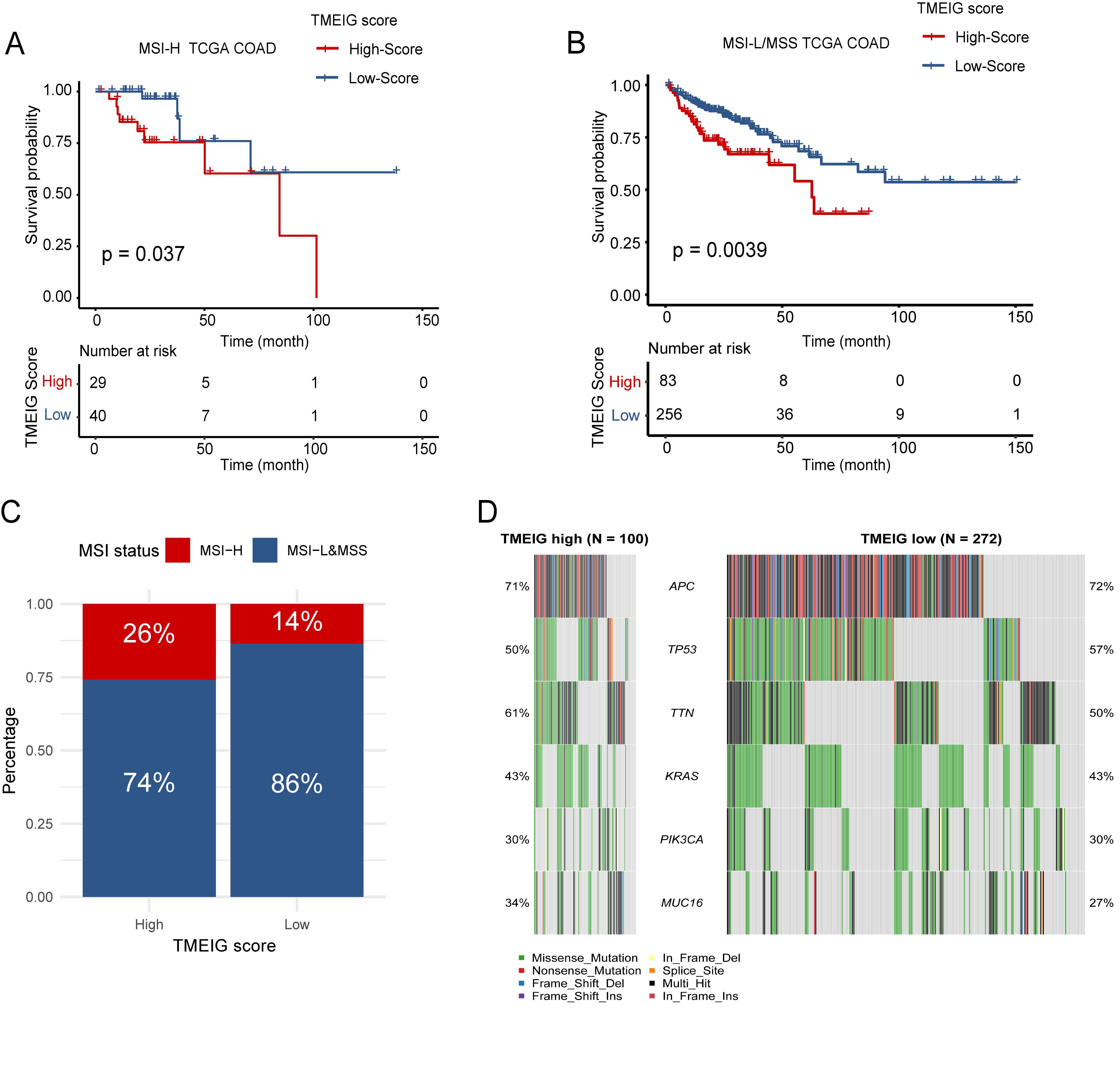

Supplement: Supplementary file 16 [file Image5.tif]
