# Supplementary material for: Tumor Microenvironment Heterogeneity-Based Score System Predicts Clinical Prognosis and Response to Immune Checkpoint Blockade in Multiple Colorectal Cancer Cohorts
Source: Front Mol Biosci. 2022 Jun 28;9:884839. doi: 10.3389/fmolb.2022.884839 (PMC9274205; doi:10.3389/fmolb.2022.884839)
Supplement: Supplementary file 13 [file Table3.docx]

| Primary antibody dilution |  |
| --- | --- |
| Antibody | Dilution Rate |
| FABP4 | 1:200 |
| SCG2 | 1:200 |
| CALB2 | 1:200 |
| HOXC6 | 1:150 |
| PAI1 (SERPINE1) | 1:200 |
| Scoring system | |
| A staining scoring system was evaluated by both staining intensity (negative=0, weak=1, and strong=2) and staining area (<5%=0, 5%-25%=1, 25%-50%=2, 50%-75%=3, and >75%=4). The staining intensity score was computed, and the score of the staining area was the final staining score. Total score<3 was considered as a weak expression. Total score>3 was considered as a strong expression. | |
| Total scores | Interpretation |
| 1,2,3 | Low expression |
| 4,5,6 | High expression |
